# Limited validity of an AI-powered app for dietary assessment in females with obesity

**DOI:** 10.1038/s41746-026-02536-2

**Published:** 2026-03-17

**Authors:** Michele Serra, Daniela Alceste, Nicole Jucker, Lotta Haupt, Sebastian Elben, Samuel Müller, Paul J. M. Hulshof, Harro A. J. Meijer, Andreas Thalheimer, Robert E. Steinert, Philipp A. Gerber, Alan C. Spector, Daniel Gero, Marco Bueter

**Affiliations:** 1 Department of Endocrinology, Diabetology and Clinical Nutrition, University Hospital Zurich, Zurich, Switzerland; 2 Department of Visceral and Transplant Surgery, University Hospital Zurich, Zurich, Switzerland; 3 Department of Health Sciences and Technology, ETH Zürich, Zurich, Switzerland; 4 Division of Human Nutrition, Wageningen University, Wageningen, The Netherlands; 5 Centre for Isotope Research (CIO), Energy and Sustainability Research Institute Groningen, University of Groningen, Groningen, The Netherlands; 6 Department of General Surgery, Hospital Männedorf, Männedorf, Switzerland; 7 Department of Psychology and Program in Neuroscience, Florida State University, Tallahassee, FL USA

**Keywords:** Health care, Medical research

## Abstract

Artificial intelligence (AI) is transforming dietary assessment, yet few tools have been clinically validated against physiological reference methods. This cross-sectional observational validation study conducted under free-living conditions evaluated the validity of SNAQ, an AI-powered image-based dietary assessment app, against doubly labelled water (DLW) in females with obesity. Twenty participants completed a 7-day protocol, including DLW-based measurement of total daily energy expenditure (TDEE) and estimation of total daily energy intake using SNAQ and 24-h dietary recall (24HR). Compared with DLW-derived TDEE (3004 ± 481 kcal/day), SNAQ underestimated energy intake by 25% (bias −817 kcal/day; limits of agreement −3707 to 2073 kcal/day), while 24HR underestimated intake by 50%. Individual-level agreement had negligible within-subject reliability (ICC = 0.00). Despite advanced AI architecture, SNAQ showed systematic group-level underestimation and poor individual-level agreement, underscoring the translational gap between algorithmic performance and clinical feasibility and the need for standardised clinical validation before implementation.

## Introduction

Patient-facing mobile health applications are increasingly promoted as tools to support and improve healthcare delivery, symptom monitoring, and lifestyle modification. In nutrition research and clinical care, such tools offer the potential to reduce participation burden and automate dietary assessment. However, enthusiasm is tempered by limited evidence regarding their clinical effectiveness, safety, validity, reliability, and concerns surrounding data security and privacy^1–3^. Furthermore, digital health studies frequently face challenges related to participant adherence and data quality under free-living conditions, which may compromise data validity^4–6^. Although international standards such as the CEN ISO/TS 82304-2:2021 are an attempt to address these issues^7^, concerns persist about their adequacy for diverse patient populations, including vulnerable groups^8^.

Accurate dietary assessment is central to clinical nutrition and obesity management, as it underpins therapeutic decision-making, tailoring of interventions, and monitoring of treatment response^9^. In Switzerland, dietary assessment is mandatory prior to initiating pharmacological or surgical interventions for obesity management, as indicated by the guidelines of the Swiss Multidisciplinary Obesity Society (SMOB). Traditional methods such as 24-h dietary recalls (24HR) and food frequency questionnaires are labour-intensive, interviewer-dependent, and prone to systematic underestimation of energy intake, particularly among individuals with obesity^9^. Although doubly labelled water (DLW) provides a physiological criterion measure for energy expenditure by quantifying carbon dioxide production from isotopic elimination rates under free-living conditions, its cost and complexity limit routine clinical use^10,11^.

Smartphone-based dietary assessment tools leveraging computer vision for image recognition have been proposed as a potential improvement by automating food recognition and nutrient content estimation^12–14^. However, their performance may be affected by factors intrinsic to eating behaviour in free-living conditions, including portion size variability, heterogeneous food presentations, lighting conditions, image distortion, and partial occlusion of foods. These challenges may be particularly pronounced in individuals with obesity if eating patterns, meal composition, and portion sizes differ from those represented in training datasets. Errors at the level of portion estimation or food recognition may therefore propagate into clinically relevant inaccuracies in estimated energy intake, with potential implications for nutritional counselling, obesity management, and treatment decisions.

SNAQ (not an acronym), a Swiss-based startup, developed one such mobile application that uses depth-sensing technology and computer vision to estimate energy and macronutrient intake from food pictures^15^. A previous validation study against DLW in females with normal weight suggested reasonable agreement at the group level, highlighting potential utility for clinical nutrition and research^16^. Whether this performance translates to adults with obesity under free-living conditions remains unknown.

Given the increasing prevalence of obesity and the clinical reliance on dietary assessment in obesity management, validation of AI-powered tools in this population is critical before clinical implementation. Therefore, this study aimed to evaluate the validity of SNAQ for estimating total energy intake in adult females with obesity under free-living conditions, using DLW as the reference method. Performance was further contextualised by comparison with 24HR, an established non-digital dietary assessment method. Based on prior evidence showing reduced accuracy of dietary assessment methods in individuals with obesity^9^, we hypothesised that the agreement between SNAQ-derived energy intake and DLW-derived energy expenditure would be lower under free-living conditions than previously reported in a normal-weight population^16^, with increased individual-level variability.

## Results

### Participant characteristics

Seventy-one adult females with obesity were invited to participate, 23 expressed interest and met eligibility criteria, and 20 completed the study (three were excluded: one due to viral infection, two due to protocol deviations). Participants had a mean age of 37.9 years and a mean BMI of 39.3 kg/m² (Table 1, Supplementary Table 7).

**Table 1:** Baseline characteristics of the study participants

	Cohort
	Obesity, *N* = 20^a^
*Biological sex*	
Female	20 (100%)
Age (years)	37.9 (13.4)
Height (m)	1.66 (0.06)
Weight (kg)	108.4 (16.3)
BMI (kg/m^2^)	39.3 (5.4)
Smoking^b^	6/20 (30%)
*Self-reported dietary adherence*	
None	16/20 (80%)
FODMAP diet	1/20 (5%)
Ketogenic diet	1/20 (5%)
Low-Carb diet	1/20 (5%)
Shellfish-Free diet	1/20 (5%)
*Physical activity (h/week)*	
None	10/20 (50%)
1–2 h/week	1/20 (5%)
2–6 h/week	7/20 (35%)
>6 h/week	2/20 (10%)
Basal metabolic rate (kcal)	1449.6 (114.9)

^a^Mean (SD) or Frequency (%), SD = standard deviation and % percentage.

^b^Independent of the amount of cigarettes smoked and smoking occasions.

### Quality control of isotope measurements

The dilution-space ratio of ^2^H to ^18^O (*N*_d_/*N*_o_) was 1.0398 ± 0.0067, and the elimination-rate ratio (*K*_d_/*K*_o_) was 1.3207 ± 0.0598. Inter-assay coefficients of variation for isotope abundance measurements were within the expected analytical range for off-axis integrated cavity output spectroscopy, as reported previously for DLW applications (<1% for δ^18^O and <2% for δ^2^H)^17^. Detailed individual results of the DLW analyses, dilution spaces, and elimination rates are provided in Supplementary Tables 8–10.

### Energy intake and expenditure estimates

**Fig. 1:**
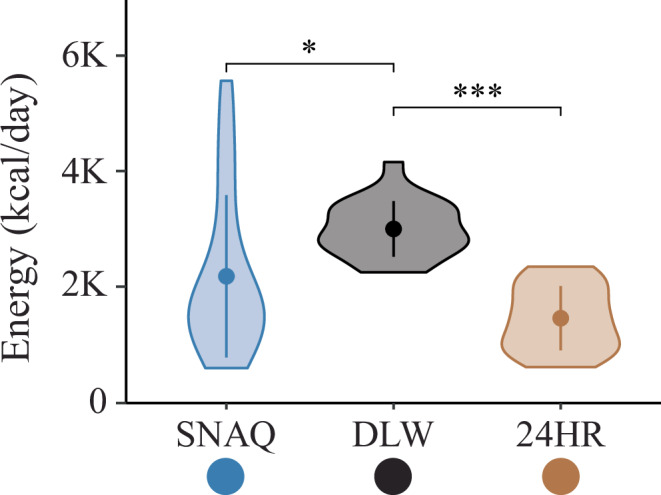
Measurement differences of total daily energy intake of the SNAQ app and the 24-hour dietary recall (24HR) in relation to the total daily energy expenditure estimated with the doubly labelled water (DLW) technique. Violin plots of the energy estimates of SNAQ, DLW, and 24HR. Means of the groups are represented with full points in the middle of the violin plots. Standard deviations are represented with a vertical line in the middle of the violin plots. Pairwise comparisons of SNAQ and DLW, and 24HR and DLW are represented with horizontal lines between the violin plots. *P*-values of the pairwise comparisons are reported above the horizontal lines. Significance levels were adjusted for multiple comparisons (3×) and are reported below the horizontal lines. They are expressed as follows: *, when *p*-value < 0·05; ***, when *p*-value < 0.001. 24HR 24-hour dietary recall, DLW doubly labelled water, *K* decimal unit suffix for thousand.

Mean TDEI estimates differed significantly between methods, with both SNAQ and 24HR yielding lower values than DLW-derived TDEE (Fig. 1, Table 2). Energy estimates from SNAQ showed greater variability and right-skewed distributions compared with 24HR and DLW (Supplementary Table 11).

**Table 2:** Energy estimates of the two dietary assessment tools and the DLW technique

Energy intake	Methods of energy estimation		
	SNAQ, *N* = 20^a^	24HR, *N* = 20^a^	DLW, *N* = 20^a^
Energy (kcal/day)	2187.0 (1400.8)	1464.0 (554.1)	3004.2 (480.5)
Energy (kJ/day)	9150.44 (5861.1)	6125.2 (2318.3)	12,569.7 (2010.4)

^a^Mean (SD), SD standard deviation.

Both SNAQ and 24HR underestimated energy intake relative to DLW, with a larger bias observed for 24HR. While SNAQ occasionally overestimated intake in a small number of participants, underestimations predominated for both methods (Supplementary Tables 12–16).

### Agreement between dietary assessments and DLW

**Fig. 2:**
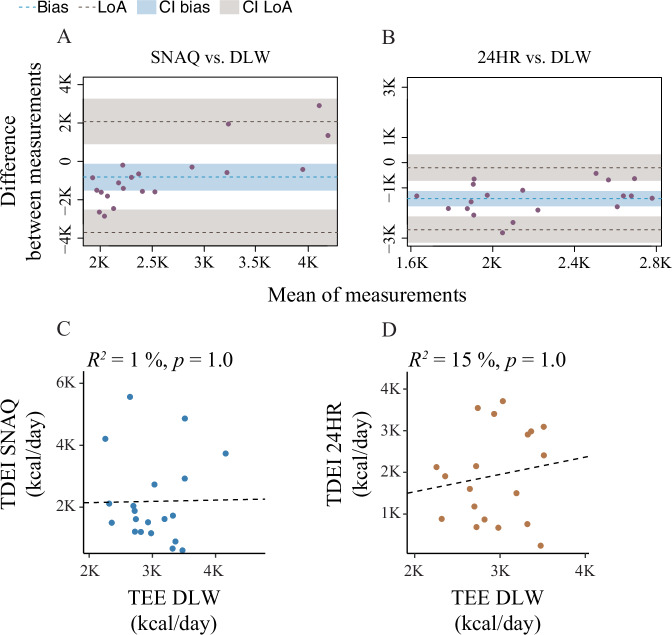
Agreement and association of the SNAQ app and the 24-hour dietary recall (24HR) with the doubly labelled water (DLW) technique. **A** Bland–Altman plot for agreement on energy intake between SNAQ and DLW. **B** Bland–Altman plot for agreement on energy intake between 24HR and DLW. The bias is represented as a blue horizontal dotted line. The value of the bias is estimated by the mean difference in energy estimation between DLW and SNAQ or 24HR. The 95% limits of agreement (LoA) are represented as two brown dotted lines and are defined as mean difference ± 1.96 standard deviations. **C** Analysis of the linear relationship between energy estimates of SNAQ and DLW. **D** Analysis of the linear relationship between energy estimates of 24HR and DLW. 24HR 24-hour dietary recall, CI confidence interval, DLW doubly labelled water, *K* decimal unit suffix for thousand, LoA limit of agreement, *p* *p*-value of the coefficient of determination, *R*^2^ coefficient of determination of the linear relationship.

Bland–Altman analyses demonstrated substantial negative bias and wide limits of agreement for both SNAQ and 24HR relative to DLW, indicating poor individual-level agreement (Fig. 2A, B, Table 3). There was no significant association between TDEE (DLW) and TDEI estimated by SNAQ or by 24HR (Fig. 2C, D).

**Table 3:** Results of the Bland–Altman plot for agreement between SNAQ and DLW, and 24HR and DLW

Agreement between	Bias	lCI bias	uCI bias	SD bias	SE bias	lLoA	uLoA	SE LoA
SNAQ and DLW	−817.3	−1507.3	−127.1	1474.5	329.7	−3707.3	2072.8	571.1
24HR and DLW	−1479.5	−1785.8	−1173.2	635.5	145.8	−2725.0	−234.0	252.5

The doubly labelled water (DLW) was selected as the reference method in the analysis.

*24HR* 24-hour dietary recall, *Bias* bias of agreement, *lCI* lower 95% confidence interval, *lLoA* lower limit of agreement, *SD* standard deviation, *SE* standard error, *uCI* upper 95% confidence interval, *uLoA* upper limit of agreement.

### Agreement between SNAQ and 24HR

**Fig. 3:**
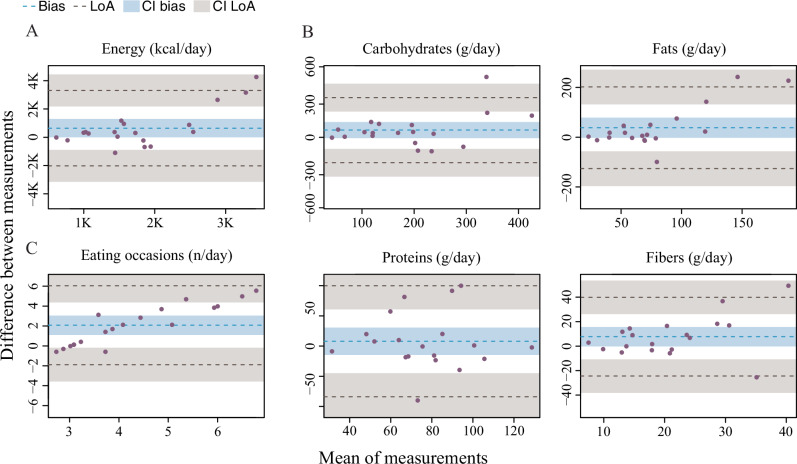
Bland–Altman plots for agreement between the SNAQ app and the 24-hour dietary recall (24HR). **A** Bland–Altman plot for total daily energy intake. **B** Bland–Altman plots for macronutrient intake. **C** Bland–Altman plot for the number of eating occasions. The bias is represented as a black horizontal line. The value of the bias is estimated by the mean difference in intake estimation between 24HR and the app for total daily energy intake and macronutrient intake. The 95% limits of agreement (LoA) are represented as two dotted lines and are defined as mean difference ± 1.96 standard deviations. CI confidence interval, *K* decimal unit suffix for thousand, LoA limits of agreement.

Compared to 24HR, SNAQ yielded higher estimates of TDEI, macronutrient intake, and number of eating occasions, with evidence of systematic bias at higher intake levels, except for protein intake (Fig. 3A, B, Table 4). Furthermore, a systemic bias could be identified in the Bland–Altman plot for the number of eating occasions at both low and high mean values (Fig. 3C).

**Table 4:** Results of the Bland–Altman plot for agreement between SNAQ and 24HR

	Bias	lCI bias	uCI bias	SD bias	SE bias	lLoA	uLoA	SE LoA
Energy (kcal)	641.7	–13.3	1296.7	1358.9	311.8	–2021.7	3305.1	540.0
Energy (kJ)	2684.8	–55.6	5425.2	5685.7	1304.4	–8458.9	13,828.5	2259.3
Carbohydrates (g)	60.8	–7.3	129.0	141.4	32.4	–216.3	338.0	56.2
Sugars (g)	38.9	–12.5	90.3	106.6	24.5	–170.2	247.9	42.4
Fats (g)	37.7	–2.7	78.1	83.8	19.2	–126.5	201.9	33.3
Saturated fats (g)	21.6	–6.4	49.6	58.1	13.3	–92.3	135.5	23.1
Proteins (g)	8.0	–14.6	30.6	46.9	10.8	–83.9	99.9	18.6
Fibres (g)	7.7	–0.2	15.6	16.4	3.8	–24.5	39.8	6.5
Dietary occasions (*n*)	2.1	1.1	3.1	2.0	0.5	–1.9	6.0	0.8

The 24-hour dietary recall (24HR) was selected as the reference method in the analysis.

*Bias* bias of agreement, *lCI* lower 95% confidence interval, *lLoA* lower limit of agreement, *SD* standard deviation, *SE* standard error, *uCI* upper 95% confidence interval, *uLoA* upper limit of agreement.

### Classification of reporting accuracy using Goldberg cut-offs

Across all tested combinations of energy estimates and physical activity estimation methods (Supplementary Tables 17–28), the classification yielding the highest proportion of plausible reporters was obtained when aBMR was combined with PAL calculated as TDEE/REE and the *S* factor specific to the study population (*S* = 40.01%, CVwTDEI = 88.36%, CVtP = 16.8%; 95% CI: 1.74–2.49). This approach identified six plausible reporters for SNAQ and five for the 24HR. Accordingly, ~30% of TDEI estimates were classified as plausible reports (Supplementary Table 29).

### Relationship between body composition, energy estimates, and body weight changes

**Fig. 4:**
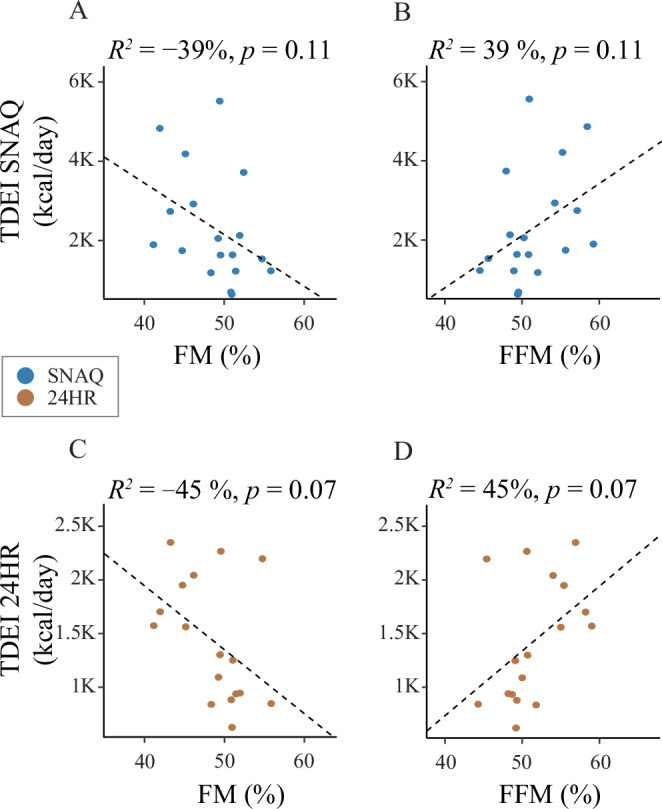
Linear models of the relationship between body composition (fat mass, FM and fat-free mass, FFM) and the total daily energy intake (TDEI) estimated with the SNAQ app and the 24-hour dietary recall (24HR). **A** Linear model of the relationship between TDEI estimated with SNAQ and FM. **B** Linear model of the relationship between TDEI estimated with SNAQ and FFM. **C** Linear model of the relationship between TDEI estimated with 24HR and FM. **D** Linear model of the relationship between TDEI estimated with 24HR and FFM. 24HR 24-hour dietary recall, *p* *p*-value of the coefficient of determination, *R*^2^ coefficient of determination of the linear model.

There were no significant correlations between energy estimates (SNAQ or 24HR) and body composition parameters (FFM, FM), nor between changes in body weight and differences in energy estimation methods (Fig. 4).

### Stability of dietary reporting across the study week

Dietary intake reporting via SNAQ showed no stability over the study week (intraclass correlation coefficient [ICC] = 0.00, 95% CI: −0.083, 0.168; *p* = 0.47).

### Comparison between SNAQ and 24HR

Despite no significant overall difference in TDEI, considerable individual variability existed. SNAQ estimated significantly more daily eating occasions (Supplementary Table 14). Energy intake from macronutrients showed no significant overall differences but large individual variations.

### Comprehensive validity assessment of SNAQ

The comprehensive evaluation indicated limited validity of SNAQ for dietary assessment in adult females with obesity, highlighting significant biases, weak correlations with energy expenditure, frequent misreporting, absence of correlation with body composition or energy storage changes, and no stability in daily reporting (Table 5). All data and results are available in the Supplementary Information.

**Table 5:** Comprehensive analysis of the validity of SNAQ for assessment of total daily energy intake in adult females with obesity

Reference method DLW		New assessment tool SNAQ for Obesity	Established assessment tool 24HR for Obesity	New assessment tool SNAQ for Normal Weight
(1)	*Agreement* Bland–Altman plots	−817.3 kcal/day LoA: −1507.3 to −127.1	−1479.5 kcal/day LoA: −1785.8 to 1173.2	−329.6 kcal/day LoA: −1503.8 to −844.5
(2)	*Strength of relationship* Pearson’s correlation coefficient	*R*^2^ = 1%, *p* = 1.0	*R*^2^ = 15%, *p* = 1.0	*R*^2^ = 27%, *p* = 0.5
(3)	*Accuracy* Absolute and percentage difference	−817.3 ± 1474.5 kcal/day (−25.0%) *p* = 0.01	−1479.5 ± 635.5 kcal/day (−49.7%) *p* < 0.0001	−329.6 ± 559.0 kcal/day (−12.8%) *p* = 0.07
(4)	*Misreporting* Over- and under-estimations	+2072.3 kcal/day (+78.4%) −1327.1 kcal/day (−43.2%)	No overestimation −1479.5 kcal/day (−49.7%)	+391.5 kcal/day (+19.8%) −591.9 kcal/day (−24.5%)
(5)	*Misreporting* Goldberg cut-off points	6 plausible reporters, 4 over-reporters 8 under-reporters	5 plausible reporters 0 over-reporters 12 under-reporters	6 plausible reporters, 5 over-reporters 19 under-reporters
(6)	*Homoeostatic control* Relationship with fat-free mass	*R*^2^ = −21%, *p* = 0.74	*R*^2^ = 14%, *p* = 1.0	*R*^2^ = −50%, *p* = 0.01
(7)	*Energy balance* Relationship with energy storages	*R*^2^ = −25%, *p* = 0.58	*R*^2^ = 6%, *p* = 1.0	*R*^2^ = 38%, *p* = 0.08
(8)	*Compliance* Relationship of measurement difference	*R*^2^ = −34%, *p* = 0.28	*R*^2^ = −27%, *p* = 0.54	*R*^2^ = −38%, *p* = 0.08
(9)	*Diet stability* Intraclass correlation coefficient	0%	NA	27%

The nine facets of validity included in the analysis have been introduced in a previous work^16^.

## Discussion

This observational study investigated under free-living conditions the validity and reliability of an AI-powered, image-based dietary assessment tool (SNAQ) in adult females with obesity. By comparing its estimates against two established reference methods (DLW and 24HR), we found substantial discrepancies that question the readiness of current AI-powered nutrition technologies for clinical or research application in populations affected by obesity.

The key finding of our investigation was the pronounced underestimation of energy intake by SNAQ relative to DLW (bias: −817.3 kcal/day; −25%). While underreporting is a known limitation in dietary assessment, the magnitude and variability observed here raise specific concerns about the viability of automated, computer-vision-based approaches in obesity research and care. Importantly, the observed underestimation likely reflects a combination of user-related factors, such as incomplete or inconsistent photo capture in free-living settings, and algorithm-related limitations, including food misclassification, portion-size estimation errors, and partial occlusion of foods within images.

In addition, while SNAQ is trained to recognise and quantify both solid foods and beverages, including drinks in packaging and in cups or glasses, differential performance between liquid and solid food items in laboratory settings or under free-living conditions has not been systematically evaluated in the literature. Given that energy from beverages, particularly sugar-sweetened drinks, may contribute substantially to total intake, uncertainty in liquid food quantification could further contribute to misalignment between estimated intake and energy expenditure.

Although group-level estimates from SNAQ did not significantly differ from 24HR, this apparent agreement masked large individual errors. On average, the mean intake estimated by SNAQ (2187.0 kcal/day) was numerically closer to DLW (3004.2 kcal/day) than 24HR (1464.0 kcal/day), but this reflected a few large overestimations offsetting underreporting rather than true accuracy. Consequently, the closer alignment of SNAQ with DLW at the group level does not translate into higher validity at the individual level but rather high variability, limiting its precision.

The underestimation of dietary intake is likely even greater than calculated, as DLW may slightly underestimate true total energy expenditure (usually by <5% and up to 10% at very high fat mass) because a small portion of deuterium may be retained in newly synthesised fat rather than lost as water^18–20^. Nevertheless, DLW remains the most accurate reference under free-living, weight-stable conditions, including in obesity^21^, and it can be assumed that it remains highly accurate under the current experimental conditions^22^.

The isotope dilution (*N*_d_/*N*_o_ = 1.04) and elimination ratios (*K*_d_/*K*_o_ = 1.32) fell within physiological ranges reported in prior human validation studies of the DLW method^19,23^, confirming valid DLW kinetics for water and CO_2_ fluxes expected in free-living adults. Agreement between body-composition estimates derived from bioelectrical impedance and isotope dilution was also within acceptable limits (Supplementary Table 30).

Our analysis shows that methodological choices for estimating energy requirements markedly influence plausibility classification under the Goldberg framework. Using Black’s generalised variability parameters^24^, none were plausible reporters, whereas applying an *S* factor specific to our cohort increased plausibility. Therefore, plausibility strongly depends on assumed within-person variability rather than necessarily reflecting true accuracy. Because aBMR is lower than REE, the TDEI:BMR ratio increases while the PAL (TDEE:REE) decreases, artificially shifting observations toward plausibility. At present, no consensus defines which combination of estimation methods best represents physiological reality, underscoring the need for empirical calibration. Further work combining direct calorimetry and body-composition-based modelling will be needed to define physiologically justified boundaries for plausibility assessment.

Our findings are consistent with previous studies reporting significant inaccuracies in digital dietary assessment tools, particularly in populations with obesity^14,25^. Collectively, this body of evidence suggests a systemic limitation of current digital methods to accurately quantify dietary intake in free-living conditions. Such limitations have important clinical implications, as imprecise dietary data may undermine nutritional counselling, obesity management, and research.

In this context, an average underestimation of energy intake of ~25% appears clinically relevant but cannot be interpreted against a single acceptability threshold, as no consensus defines such cut-offs in clinical nutrition. This highlights the limitation of relying on isolated metrics and the need for multidimensional validity frameworks that integrate bias, variability, physiological plausibility, and stability over time, together with clinical oversight by trained professionals when interpreting AI-powered dietary assessments.

This interpretation is supported by prior DLW-based validation studies of image-assisted dietary assessment. In the study by Most et al., dietary intake was assessed using the SmartIntake application based on the remote food photography method (RFPM), in which food identification and portion-size estimation were performed by trained and certified human raters using a standardised food image library, rather than by trained AI^14^. In that study, mean reported intake corresponded to ~63.4% of DLW-derived TDEE, equivalent to an average underestimation of 35.6%, with relatively low variability (SEM ± 3.2%, *n* = 23). In contrast, despite a comparable sample size (*n* = 20), SNAQ yielded substantially greater dispersion in relative error (SD ± 51.1%, SEM ± 11.4%). This difference in variability cannot be attributed to sample size alone and instead suggests that full automation of food recognition and portion estimation introduces additional sources of measurement instability at the user-technology interface. Increasing sample size would reduce uncertainty around the mean estimate but would not resolve the pronounced individual-level variability observed here.

In a parallel comparison within our own work (Table 5), bias was notably larger in adult females with obesity compared with the previous cohort with normal weight (−817.3 vs. −329.6 kcal/day), despite the use of the same AI-powered assessment system. The reasons for this difference cannot be explained with the present data and may reflect complex interactions between physiology, free-living context, and human–technology interaction rather than properties of the algorithm alone. Importantly, this uncertainty underscores the value of multidimensional validity benchmarks, which allow systematic identification of where and how performance decreases across populations without relying on speculative attribution to behavioural or physiological factors. Within these interpretive constraints, the absence of linear associations between reported intake, energy expenditure, and body-composition variables, together with negligible reproducibility (ICC = 0), indicates poor physiological plausibility and instability at the individual level of dietary records collected with SNAQ.

We also observed occasional marked overestimations by SNAQ, likely caused by user–AI interaction errors such as mis-captured portions or database mismatches. Such bidirectional errors could lead to inappropriate clinical recommendations, reinforcing the need for rigorous validation before use.

Because errors in energy estimation propagate to macro- and micronutrients, inaccurate total energy intake compromises nutritional assessment and therapy, especially in obesity management. This underlines the urgency for comprehensive validation of digital dietary tools.

Our evaluation aligns with broader concerns about premature adoption of unvalidated AI health technologies. Analogous failures, such as the Epic Sepsis Model, show the risks of integrating untested algorithms into care. Digital interventions improve outcomes only when supported by evidence of accuracy, safety, and efficacy^26–28^.

Despite their operational appeal, automated nutrition systems remain vulnerable to major inaccuracies when not peer-reviewed and clinically validated. Economic incentives for rapid deployment must not outweigh patient-safety considerations.

Strengths of this study include the use of DLW as a reference and a homogeneous cohort of adult females with obesity without comorbidities, minimising confounders. Limitations include small sample size and absence of structured adherence monitoring, preventing clear attribution of user- versus technology-related error. Incomplete photographic logging may have contributed to underestimation, reinforcing composite errors at the human–AI interface.

While technology readiness is limited, patient readiness also matters: effective integration will require improved adherence, literacy, and engagement without attributing bias to users or reinforcing stigma. The observed underestimation of energy intake might also arise from the challenges users face when integrating such technologies into daily life. In addition, concerns related to data security and privacy, particularly when image-based dietary data are collected and stored in free-living conditions that capture aspects of private daily life, may further affect user trust and willingness to engage with AI-powered assessment tools. Generalisability of these findings should therefore be interpreted cautiously, as factors such as ethnicity, socioeconomic status, and smartphone literacy may further moderate tool validity through differences in dietary patterns, food presentation, access to technology, and correct tool use. Effective implementation of AI-powered technologies into clinical workflows will therefore require not only methodological validation and technical refinement, but also strategies to enhance user adherence, literacy, acceptance, and trust.

Classification of reporting plausibility by the Goldberg method should be interpreted cautiously, as predictive equations (not direct measures) were used for energy requirements. Advanced models based on organ-tissue composition (e.g., MRI) could refine these estimates, though even such methods achieve <60% accuracy in obesity^29,30^. Thus, current plausibility cut-offs remain method-dependent.

Potential database discrepancies between SNAQ and Nutritics likely explain <10% of energy-estimate variance^31,32^ and cannot account for the observed bias, consistent with our previous validation for adult females with normal weight.

Future studies combining physical-activity tracking and repeated recalls could strengthen reliability, but were beyond the scope of this study.

Developers of AI-powered nutrition tools should be held to explicit analytical-validity standards before clinical or research release. We propose establishing a benchmarking framework, analogous to diagnostic-device performance criteria, defining allowable bias, limits of agreement, and intra-individual reliability.

Although the present study cannot establish such standards, it illustrates their necessity and feasibility. Extending our previously introduced quantitative indicators into a unified benchmark could enable within- and between-population evaluation. Development should be coordinated by professional societies (EFAD, ASN, IUNS) together with digital-health standardisation bodies, to set measurable targets and prevent premature deployment of unvalidated tools. Establishing such a consensus-driven framework would provide developers with measurable performance targets, promote methodological transparency, and safeguard clinical nutrition practice from the premature deployment of inadequately validated technologies.

Future research must generate the evidence to populate such benchmarks through large, diverse, longitudinal validations and analyses of user- and algorithm-driven errors. Both healthcare systems and developers share responsibility for ensuring that digital nutrition technologies meet these standards before implementation.

In conclusion, the current generation of AI-powered dietary assessment apps, exemplified by SNAQ, lacks validity for precise dietary monitoring in adult females with obesity. Substantial inaccuracies and instability limit their clinical implementation. Digital health innovations must undergo rigorous validation to ensure they enhance patient care rather than compromise it.

## Methods

### Study design and participants

In this cross-sectional observational study, we evaluated the validity of the image-based dietary assessment app SNAQ in adult females with obesity (BMI ≥ 30.0 kg/m²) under free-living conditions. Participants were recruited from the University Hospital Zurich between September 2020 and January 2023 within an ongoing cohort investigating dietary behaviour after Roux-en-Y gastric bypass (RYGB).

Ethical approval was obtained from the Cantonal Ethics Committee Zurich (BASEC-No. 2019-00952). Written informed consent was obtained from all participants. The study complied with the Declaration of Helsinki and is registered at ClinicalTrials.gov (NCT04600596; registered on October 23, 2020). Reporting follows STROBE-nut guidelines (Supplementary Table 1).

### Inclusion and exclusion criteria

Adult females aged ≥18 years with BMI ≥ 30.0 kg/m² proficient in smartphone use were eligible. Exclusion criteria included prior bariatric surgery, gastrointestinal and metabolic diseases affecting diet, diabetes mellitus (type 1 or 2), medications influencing appetite or metabolism, pregnancy or breastfeeding, medically prescribed restrictive diets that substantially constrain energy intake or eating behaviour, kidney or heart failure, malabsorption syndrome, mental disorders (e.g., eating disorders), and inability to understand instructions in either German or English. Detailed inclusion and exclusion criteria are provided in Supplementary Table 2. Female participants were selected owing to their predominance in our clinic (76%) and established sex differences in dietary behaviour. Dietary patterns that restrict food types but do not impose a prescribed reduction in total energy intake or eating frequency (e.g., low-carbohydrate, ketogenic, FODMAP, or food-avoidance diets without medical indication) were not considered exclusionary.

### Sample size calculation

Given the exploratory nature and absence of established validation thresholds, a target sample of 23 was chosen, consistent with prior validation studies of dietary tools^14^.

### Study objectives

The primary objective was to assess agreement between TDEI from SNAQ and TDEE from DLW. Secondary objectives were the comparison of SNAQ, DLW, and 24HR for accuracy, misreporting (Goldberg cut-offs), and associations with body composition and weight change.

### Study procedures

Participants attended two visits 8 days apart (“study week”). At baseline (day 1), anthropometry, body composition (bioelectrical impedance analysis, BIA; Seca mBCA 515), and 24HR of the previous day were collected. Participants ingested an individualised DLW dose (1.8 g/kg total body water of 10% ¹⁸O-labelled water, 0.12 g/kg TBW of 99.9% ²H-labelled water) with pre- and post-dose urine sampling (0, 4, 5 h). The specific doses administered to each participant and corresponding batch information are reported in Supplementary Tables 3 and 4. Participants logged dietary intake with the SNAQ app over the following 7 days, using smartphones provided if necessary. Semi-customised prompts at meal times minimised reporting bias. Participants avoided strenuous physical activity during the study week. At visit two (day 8), body weight was remeasured, and final urine samples were collected. Participants received a reimbursement of CHF 20 to cover travel expenses; no additional financial compensation or incentives were provided.

### Anthropometric and body composition measurements

Body weight was assessed to the nearest 0.1 kg using a Seca mBCA Ultra device (mBCA 555 platform with mBCA 550 handrail, Seca GmbH, Hamburg, Germany) after a 16-h overnight fast. Participants stood barefoot and lightly clothed on the platform while holding the integrated hand electrodes to ensure optimal skin–electrode contact. The system combines multifrequency BIA with an ultrasound-based height module (seca 257 [100–220 cm], Seca GmbH). Stature was measured without shoes to an accuracy of 0.01 m. According to manufacturer validation, the accuracy for estimating fat mass, fat-free mass, and muscle mass is 98%, 98%, and 97%, respectively. All measurements were obtained following the manufacturer’s standard operating procedure to ensure reproducibility. Waist circumference was measured manually to the nearest 0.01 m, positioned just above the anterior superior iliac spine. Baseline bioelectrical impedance vector characteristics of the study population are presented in Supplementary Table 6.

### Total daily energy expenditure measurement

TDEE was assessed using a two-point DLW method in urine over 7 days. Each participant was given a dose mixture composed of 1.8 g of 10 atom% 18oxygen-(^18^O)-labelled water (H_2_^18^O, Cambridge Isotopes Laboratories, Inc., Tewksbury, MA, USA) per kg of body water and 0.12 g of 99.9 of atom% deuterium-(^2^H)-labelled water (^2^H_2_O, Cambridge Isotopes Laboratories, Inc., Tewksbury, MA, USA) per kg of body water. Urine samples were analysed for ^2^H and ^18^O isotopic abundance at the Centre for Isotope Research, University of Groningen, The Netherlands, using an optical spectrometer (LGR LWIA 912-0050, ABB Ltd., Los Gatos Research, USA). Isotope analysis, including correction for memory effects and instrumental drift, followed established optical spectroscopy protocols^17^. Calibration was performed using International Atomic Energy Agency (IAEA) reference waters (IAEA-607, -608, -609), which were interleaved with study samples for multi-point calibration and quality control.

Carbon dioxide production (rCO₂) was computed from isotopic elimination rates (¹⁸O and ²H) and TBW^33^: rCO_2_ = 0.4664 * *N* [(1.007*k*_O_)–(1.043*k*_d_)] * 22.26. TDEE was derived from rCO_2_ using the Weir equation^34^: TDEE = rCO_2_ * (1.106 + 3.94/RQ). This conversion requires an assumption regarding the respiratory quotient (RQ), defined as the ratio of CO_2_ production to O_2_ consumption, which reflects the relative contribution of carbohydrate, fat, and protein oxidation. In accordance with standard practice in DLW studies and assuming mixed macronutrient oxidation under habitual dietary conditions, a standard RQ of 0.85 was applied^33^. This value lies within the physiological range (0.718 for fats, 0.802 for proteins, 1.0 for carbohydrates) and is commonly used when individual RQ or food quotient data are not available.

### Estimation of energy requirements and physical activity level

Energy requirements at rest were estimated using both basal metabolic rate (BMR) and resting energy expenditure (REE) to enable cross-validation and ensure methodological consistency in the application of the Goldberg cut-offs^24^. The Mifflin–St Jeor and Harris–Benedict equations were used to calculate BMR with adjusted body weight, accounting for obesity-related biases in standard equations^35^. Indeed, for adults living with obesity, BMR might be overestimated due to the lower metabolic rate of adipose tissue compared to lean tissue. Therefore, adjusted body weight was used for this study population in the equation. The adjusted body weight (ABW) was calculated with the ideal body weight (IBW) as follows: ABW = IBW + 0.3 * (weight (kg)–IBW), where the IBW for adult females was calculated according to the Devine formula^36^: IBW = 50 kg + 2.3 kg * ((height in cm–152.4)/2.54). (2) REE was calculated with Seca Analytics 125 using a proprietary regression model based on impedance-derived body composition. All estimates of energy requirements were expressed in kcal/day. The individual calculations of BMR (using actual and adjusted body weights) and REE estimates are provided in Supplementary Table 5.

Physical activity level (PAL) was estimated using three complementary approaches. First, PAL was calculated as the ratio of DLW-derived TDEE to adjusted BMR (aBMR) according to the Mifflin–St Jeor equation. Second, PAL was calculated as the ratio of TDEE REE derived from Seca Analytics 125. Third, PAL was estimated based on self-reported lifestyle categories using the proprietary algorithm implemented in Seca Analytics 125, for which calculation details are not publicly disclosed by the manufacturer.

### Dietary assessment methods

For the 24HR, trained investigators conducted structured interviews, documenting food, beverage, and supplement intake with standardised household measures. Data analysis used Nutritics software (version 5.77, 2021, Nutritics, Dublin, Ireland) for energy and macronutrient calculation. For the SNAQ app, participants logged food intake via before-and-after meal images. The app used depth-sensing technology and computer vision to estimate portion size, energy, and macronutrients, utilising proprietary and Swiss Food Composition databases.

### Definition of eating occasions

For analyses, an eating occasion was operationalised as follows:

(i) for SNAQ any recording with a pre‑meal entry in SNAQ constituted one eating occasion, irrespective of whether the corresponding post‑meal image was recorded as a completed meal or leftovers, or if it was not uploaded;
(ii) for 24HR each discrete intake entry recorded in the 24HR constituted one eating occasion.

For both dietary assessment methods, eating occasions were defined independently of clock time, energy content, food or beverage type, and duration.

### Misreporting assessment

Dietary misreporting (under- or over-reporting) was evaluated using Goldberg cut-off points^24^, comparing reported TDEI (SNAQ and 24HR) to calculated energy requirements, adjusted for variability in intake and physical activity. The variability factor *S* was calculated using two parameter sets: (i) study-specific within-subject coefficient of variation for TDEI reported via SNAQ (CV_wTDEI_) and between-subject variation of PAL (CV_tP_) and (ii) default values recommended by Black for adult females (26% and 14.9%, respectively)^24^. No published data are available for CV_wTDEI_ in adult females with obesity. In this study, CV_wTDEI_ and CV_tP_ quantified intra- and inter-individual variability in energy intake and physical activity, expressed as percentages of the mean. These coefficients are standard measures of self-report reliability. Further methodological details are provided in our previous publication^16^.

The alternative PAL estimates were used to systematically assess the classification of plausible, under-, and over-reporters across all combinations of energy requirement (aBMR or REE), PAL estimation method, and dietary assessment tool (SNAQ and 24HR), using both a study-specific variability factor and the reference values proposed by Black^24^.

### Comprehensive validity assessment

A comprehensive validity assessment was conducted in accordance with our previously proposed benchmark for the standardised evaluation and reporting of novel dietary assessment tools^16^. This framework integrates multiple complementary statistical approaches, including agreement, association, relative accuracy, misreporting, physiological plausibility, and temporal stability, to enable a multidimensional evaluation of dietary assessment validity.

### Statistical analysis

Data analysis employed R (v4.2.2) and RStudio (v2022.07.1). Bland–Altman analyses were used to assess agreement between methods, with 95% limits of agreement (LoA). Proportional (level-dependent) bias was evaluated by visual inspection of Bland–Altman plots (differences versus means). Formal regression of differences on means was not performed, as the analysis was exploratory and the primary aim was descriptive assessment of agreement rather than correction for proportional bias. Pearson’s correlation (*r*²) assessed linear associations. Differences between methods were tested via paired *t*-tests or Wilcoxon signed-rank tests based on data normality (Shapiro–Wilk). *P*-values were adjusted (Bonferroni correction) for multiple comparisons. Stability of daily intake across the study week was quantified using intraclass correlation coefficients (ICC; two-way random effects model). ICC values were interpreted according to commonly used thresholds, with values < 0.50 indicating poor reliability and values close to 0 indicating negligible reproducibility.

## Supplementary information

Supplementary Material

## Data Availability

Data described in the manuscript are available in the Supplementary Information. The de-identified individual-participant data used in this study will be made available upon request to M.S. (michele.serra@usz.ch).

## Code availability

All analyses were conducted using R (version 4.2.2). We developed an automated end-to-end analysis pipeline encompassing data preprocessing, data processing, statistical analysis, and data visualisation. All equations and computational steps described in the “Methods” section, including isotope elimination rate calculations, carbon dioxide production, and derivation of total daily energy expenditure using the Weir equation with a fixed respiratory quotient for the DLW analyses, were fully implemented within the processing stage of the pipeline. The workflow integrates raw SNAQ data with raw DLW and 24HR datasets, harmonises and merges data by study identifier, and generates all statistical outputs, tables, and figures. The pipeline requires four input datasets: raw SNAQ data, raw DLW data, 24HR-derived macronutrient intake estimates from Nutritics, and demographic data collected at study visits. The analysis code is available from M.S. upon reasonable request.
