# Supplementary material for: Limited validity of an AI-powered app for dietary assessment in females with obesity
Source: NPJ Digit Med. 2026 Mar 17;9:357. doi: 10.1038/s41746-026-02536-2 (PMC13144502; doi:10.1038/s41746-026-02536-2)
Supplement: Supplementary file 1 — Supplementary Material [file 41746_2026_2536_MOESM1_ESM.pdf]

Limited Validity of an AI-Powered App for Dietary Assessment in Females with Obesity

Michele Serra[1,2,†], Daniela Alceste[2,†], Nicole Jucker[1], Lotta Haupt[3], Sebastian Elben[3], Samuel Müller[3], Paul J M Hulshof[4], Harro A J Meijer[5], Andreas Thalheimer[6], Robert E Steinert[2], Philipp A Gerber[1], Alan C Spector[7], Daniel Gero[2], Marco Bueter[6,*]

[1]Department of Endocrinology, Diabetology and Clinical Nutrition, University Hospital Zurich, Zurich, Switzerland

[2]Department of Visceral and Transplant Surgery, University Hospital Zurich, Zurich, Switzerland

[3]Department of Health Sciences and Technology, ETH Zürich, Zurich, Switzerland

[4]Division of Human Nutrition, Wageningen University, Wageningen, The Netherlands

[5]Centre for Isotope Research (CIO), Energy and Sustainability Research Institute Groningen, University of Groningen, Groningen, The Netherlands

[6]Department of General Surgery, Hospital Männedorf, Männedorf, Switzerland

[7]Department of Psychology and Program in Neuroscience, Florida State University, Tallahassee, FL, USA

† Contributed equally

* Corresponding author

Correspondance to:

Prof Marco Bueter PhD, Department of General Surgery, Hospital Männedorf, Switzerland.

marco.bueter@uzh.ch

Supplementary Material

Supplementary figures 5

Supplementary Figure 1: Analysis of normal distribution of the data 5

Supplementary Figure 2: Bland-Altman plots for agreement between the app and the 24HR 6

Supplementary Figure 3: Measurement differences of total daily energy intake estimated with the SNAQ app and the 24-hour dietary recall (24HR) in relation to the doubly labelled water (DLW) technique 7

Supplementary Figure 4: Absolute values of total daily energy intake 8

Supplementary Figure 5: Boxplots of the total daily energy intake 9

Supplementary Figure 6: Percentage of the measurement differences of total daily energy intake 10

Supplementary Figure 7: Changes in body weight during the study period and correlation analysis of linear models of relationship between measurement differences of SNAQ and the 24-hour dietary recall (24HR). 11

Supplementary Figure 8: Linear model of the relationship between daily energy intake estimated with SNAQ and daily energy intake estimated with the 24-hour dietary recall (24HR) 13

Supplementary tables 14

Supplementary Table 1: Reporting of the study according to the STROBE-nut checklist table. 14

Supplementary Table 2: Eligibility criteria 21

Supplementary Table 3: Doses of doubly labelled water (DLW) for each study participant and relative DLW batch 22

Supplementary Table 4: Description of the composition of the three doubly labelled water batches 23

Supplementary Table 5: Calculations of BMR performed using both the actual and adjusted patient body weights according to the equations of both Mifflin-St Jeor and Harris-Benedict and estimates of Resting Energy Expenditure (REE) calculated with seca analytics 125 using a proprietary regression model based on impedance-derived body composition. 24

Supplementary Table 6: Baseline bioelectrical impedance vector analysis of the study population 25

Supplementary Table 7: Nationality of the study population 26

Supplementary Table 8: Results of the doubly labelled water (DLW) analysis 27

Supplementary Table 9: Dilution spaces of the isotopes deuterium and 18-oxygen for each study participant .. 28

Supplementary Table 10: Elimination rates of the isotopes deuterium and 18-oxygen for each study participant 29

Supplementary Table 11: Results of the Shapiro–Wilk test for normality of the distribution of the energy estimates of the SNAQ app, the 24-hour dietary recall, and the DLW technique 30

Supplementary Table 12: Absolute and percentage measurement differences of total daily energy intake estimated with the SNAQ app in relation to total daily energy expenditure estimated with DLW technique 31

Supplementary Table 13: Absolute and percentage measurement differences of total daily energy intake estimated with the 24-hour dietary recall in relation to estimates with the DWL technique 32

Supplementary Table 14: Comparison of energy intake, macronutrient intake, and eating occasions between SNAQ and 24HR 33

Supplementary Table 15: Overall difference, and over- and underestimations of total daily energy intake estimated with SNAQ and 24HR in relation to DLW 34

Supplementary Table 16: Over- and underestimations of energy and macronutrient intake estimated with SNAQ in relation to 24HR 35

Supplementary Table 17: Classification of study participants as plausible, over- or under-reporters of total daily energy intake estimated with SNAQ according to the Goldberg cut-off method,[1] using adjusted Basal

Metabolic Rate (BMR) estimated by the Mifflin-St Jeor equation and Physical Activity Level (PAL) calculated as a ratio of DLW-based estimates of total daily energy expenditures (TDEE) and BMR. **36**

Supplementary Table 18: Classification of the study participants as plausible, over- or under-reporters of total daily energy intake estimated with the 24-hour dietary recall (24HR) according to the Goldberg cut-off method,[1] using adjusted Basal Metabolic Rate (BMR) estimated by the Mifflin-St Jeor equation and Physical Activity Level (PAL) calculated as a ratio of DLW-based estimates of total daily energy expenditures (TDEE) and BMR. **37**

Supplementary Table 19: Classification of study participants as plausible, over- or under-reporters of total daily energy intake estimated with SNAQ according to the Goldberg cut-off method,[1] using adjusted Basal Metabolic Rate (BMR) estimated by the Mifflin-St Jeor equation and Physical Activity Level (PAL) selected based on lifestyle using the software seca analytics 125. .... **38**

Supplementary Table 20: Classification of study participants as plausible, over- or under-reporters of total daily energy intake estimated with the 24-hour dietary recall (24HR) according to the Goldberg cut-off method,[1] using adjusted Basal Metabolic Rate (BMR) estimated by the Mifflin-St Jeor equation and Physical Activity Level (PAL) selected based on lifestyle using the software seca analytics 125. .... **39**

Supplementary Table 21: Classification of study participants as plausible, over- or under-reporters of total daily energy intake estimated with SNAQ according to the Goldberg cut-off method,[1] using adjusted Basal Metabolic Rate (BMR) estimated by the Mifflin-St Jeor equation and Physical Activity Level (PAL) calculated as a ratio of DLW-based estimates of total daily energy expenditures (TDEE) and Resting Energy Expenditure (REE) calculated with seca analytics 125 using a proprietary regression model based on impedance-derived body composition. **40**

Supplementary Table 22: Classification of study participants as plausible, over- or under-reporters of total daily energy intake estimated with the 24-hour dietary recall (24HR) according to the Goldberg cut-off method,[1] using adjusted Basal Metabolic Rate (BMR) estimated by the Mifflin-St Jeor equation and Physical Activity Level (PAL) calculated as a ratio of DLW-based estimates of total daily energy expenditures (TDEE) and Resting Energy Expenditure (REE) calculated with seca analytics 125 using a proprietary regression model based on impedance-derived body composition. **41**

Supplementary Table 23: Classification of study participants as plausible, over- or under-reporters of total daily energy intake estimated with SNAQ according to the Goldberg cut-off method,[1] using Resting Energy Expenditure (REE) calculated with seca analytics 125 using a proprietary regression model based on impedance-derived body composition, and Physical Activity Level (PAL) calculated as a ratio of DLW-based estimates of total daily energy expenditures (TDEE) and adjusted Basal Metabolic Rate (BMR) estimated by the Mifflin-St Jeor equation. **42**

Supplementary Table 24: Classification of study participants as plausible, over- or under-reporters of total daily energy intake estimated with the 24-hour dietary recall (24HR) according to the Goldberg cut-off method,[1] using Resting Energy Expenditure (REE) calculated with seca analytics 125 using a proprietary regression model based on impedance-derived body composition, and Physical Activity Level (PAL) calculated as a ratio of DLW-based estimates of total daily energy expenditures (TDEE) and adjusted Basal Metabolic Rate (BMR) estimated by the Mifflin-St Jeor equation. **43**

Supplementary Table 25: Classification of study participants as plausible, over- or under-reporters of total daily energy intake estimated with SNAQ according to the Goldberg cut-off method,[1] using Resting Energy Expenditure (REE) calculated with seca analytics 125 using a proprietary regression model based on impedance-derived body composition, and Physical Activity Level (PAL) selected based on lifestyle using the software seca analytics 125. .... **44**

Supplementary Table 26: Classification of study participants as plausible, over- or under-reporters of total daily energy intake estimated with the 24-hour dietary recall (24HR) according to the Goldberg cut-off method,[1] using Resting Energy Expenditure (REE) calculated with seca analytics 125 using a proprietary regression model based on impedance-derived body composition, and Physical Activity Level (PAL) selected based on lifestyle using the software seca analytics 125. .... **45**

Supplementary Table 27: Classification of study participants as plausible, over- or under-reporters of total daily energy intake estimated with SNAQ according to the Goldberg cut-off method,[1] using Resting Energy Expenditure (REE) calculated with seca analytics 125 using a proprietary regression model based on

impedance-derived body composition, and Physical Activity Level (PAL) calculated as a ratio of DLW-based estimates of total daily energy expenditures (TDEE) and REE. 46

Supplementary Table 28: Classification of study participants as plausible, over- or under-reporters of total daily energy intake estimated with the 24-hour dietary recall (24HR) according to the Goldberg cut-off method,[1] using Resting Energy Expenditure (REE) calculated with seca analytics 125 using a proprietary regression model based on impedance-derived body composition, and Physical Activity Level (PAL) calculated as a ratio of DLW-based estimates of total daily energy expenditures (TDEE) and REE. 47

Supplementary Table 29: Summary of the number of plausible, under-, and over-reporters across all combinations of BMR, REE, and PAL estimation methods for the SNAQ and 24-hour dietary recall (24HR), calculated using both a study-specific S factor and the reference values proposed by Black.[1] 48

Supplementary Table 30: Results of the Bland-Altman plot for agreement between bioelectrical impedance (BIA) and isotope dilution of DLW for measurements of body composition 49

References 50

Supplementary figures

Supplementary Figure 1: Analysis of normal distribution of the data

(A) Quantile-Quantile plots of energy estimates of the SNAQ app, the 24-hour dietary recall (24HR), and the doubly labelled water (DLW) technique. The diagonal dashed line represents the perfect match between the observed (empirical) and theoretical quantiles. If the data follows the theoretical distribution, the points will closely align with this line. 95 % confidence intervals are above and below the diagonal line. (B) Density plots of the energy estimates of the three methods.

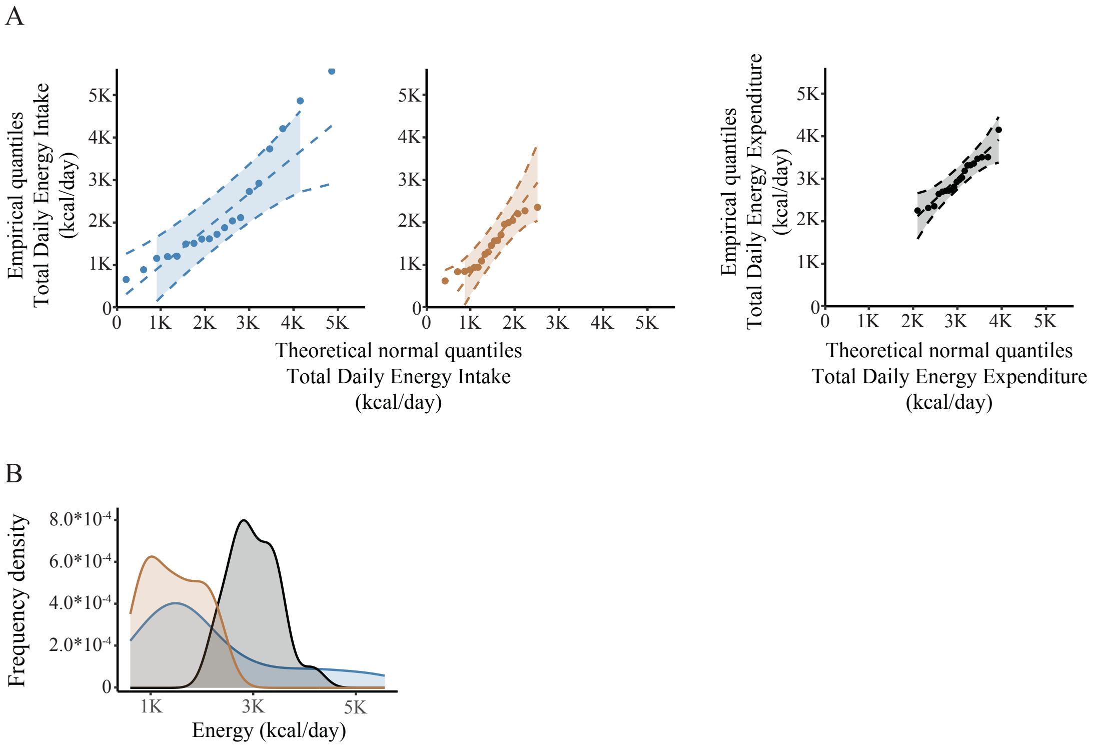

Supplementary Figure 2: Bland-Altman plots for agreement between the app and the 24HR

(A) Bland-Altman plot for sugar intake. (B) Bland-Altman plots for intake of saturated fats. The bias is represented as a black horizontal line. The value of the bias is estimated by the mean difference in intake estimation between 24HR and the app for total daily energy intake and macronutrient intake. The 95% limits of agreement (LoA) are represented as two dotted lines and are defined as mean difference ± 1.96 standard deviations.

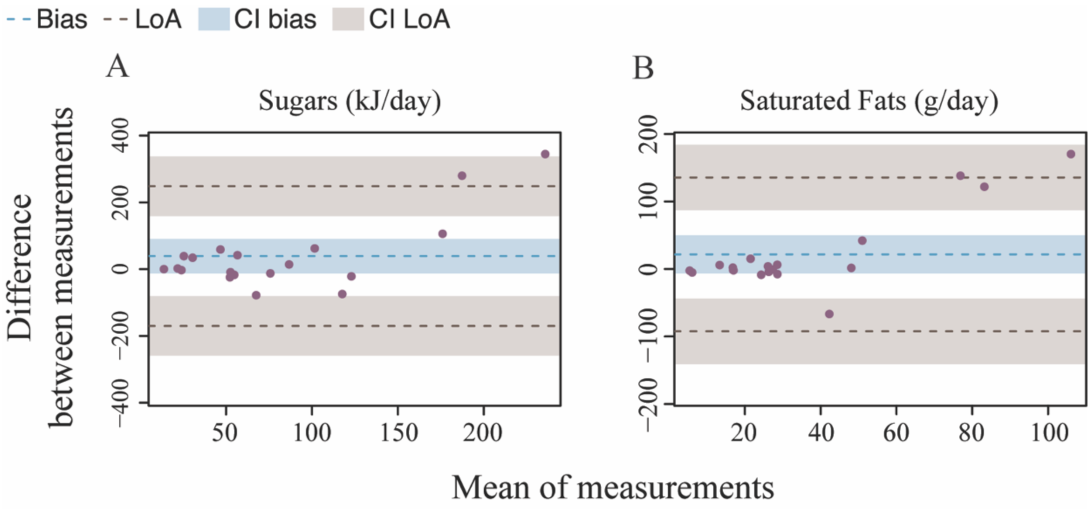

Supplementary Figure 3: Measurement differences of total daily energy intake estimated with the SNAQ app and the 24-hour dietary recall (24HR) in relation to the doubly labelled water (DLW) technique

(A) Absolute values of energy estimates with SNAQ, 24HR, and DLW. (B) Percentage of measurement differences of SNAQ and 24HR in relation to DLW. Vertical dotted red lines mark 50-% breaks to support the interpretation of the bars on the top of the panel.

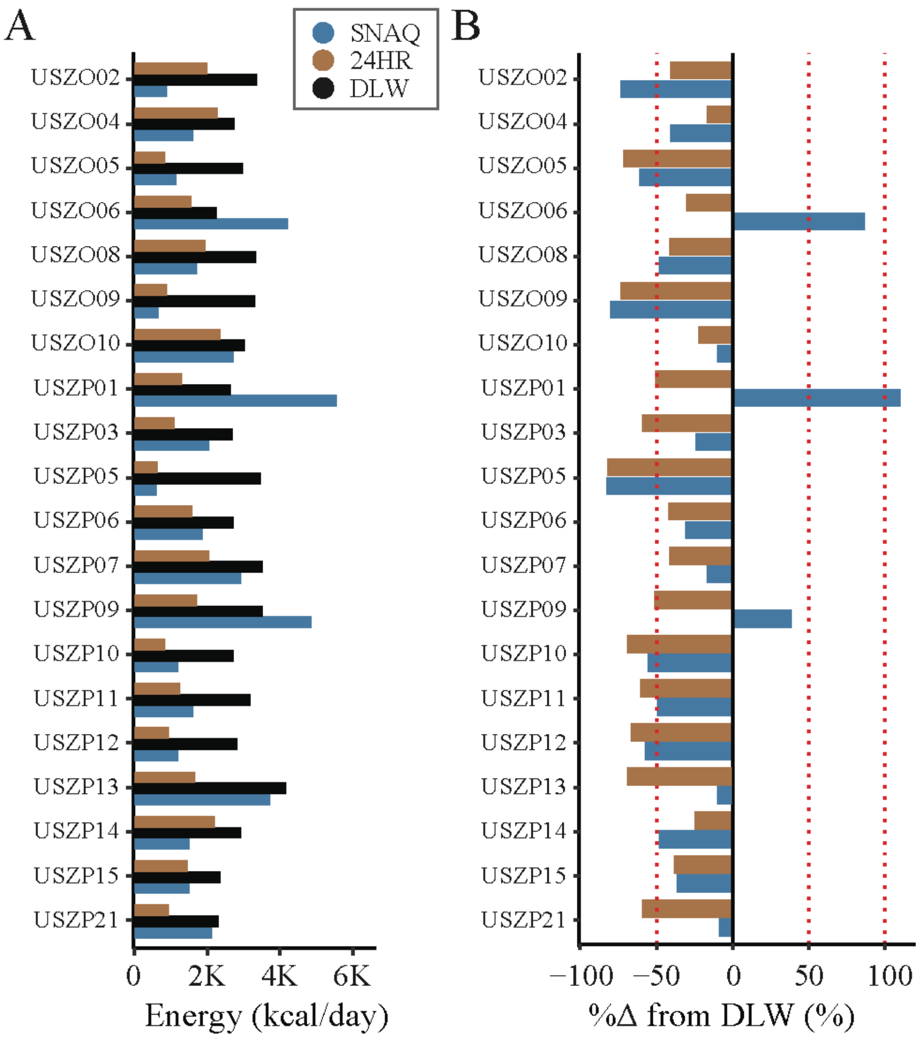

Supplementary Figure 4: Absolute values of total daily energy intake

(A) macronutrient intake (B-G) and eating occasions (H) estimated with the SNAQ app and the 24-hour dietary recall (24HR).

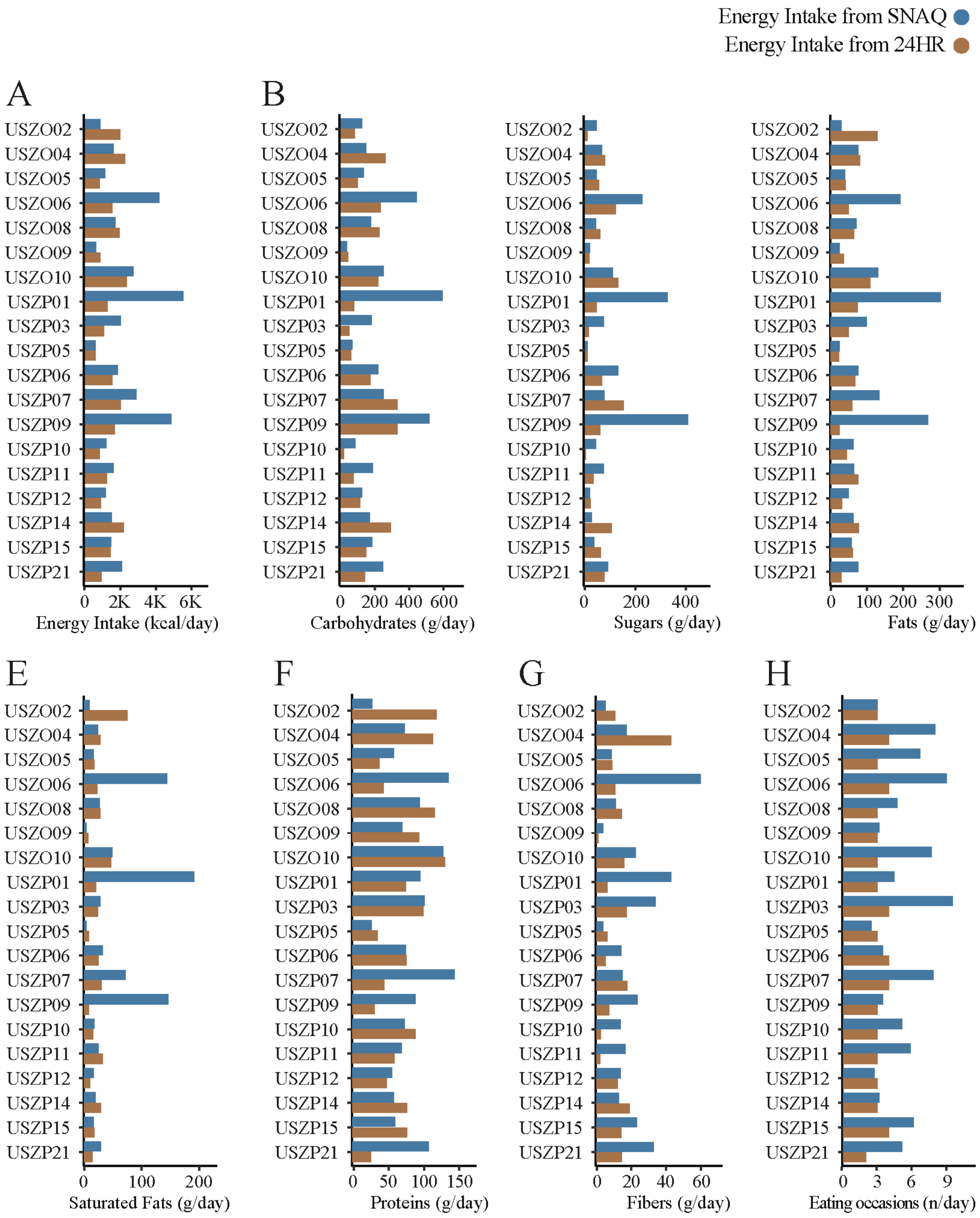

Supplementary Figure 5: Boxplots of the total daily energy intake (A) of the macronutrient intake (B-G) and eating occasions (H) estimated with the SNAQ app and the 24-hour dietary recall. Paired Student's t-test have been performed on the datasets of SNAQ and 24HR for each variable. The results of the t-tests are reported on top of each panel. Paired measurements are connected with a grey line.

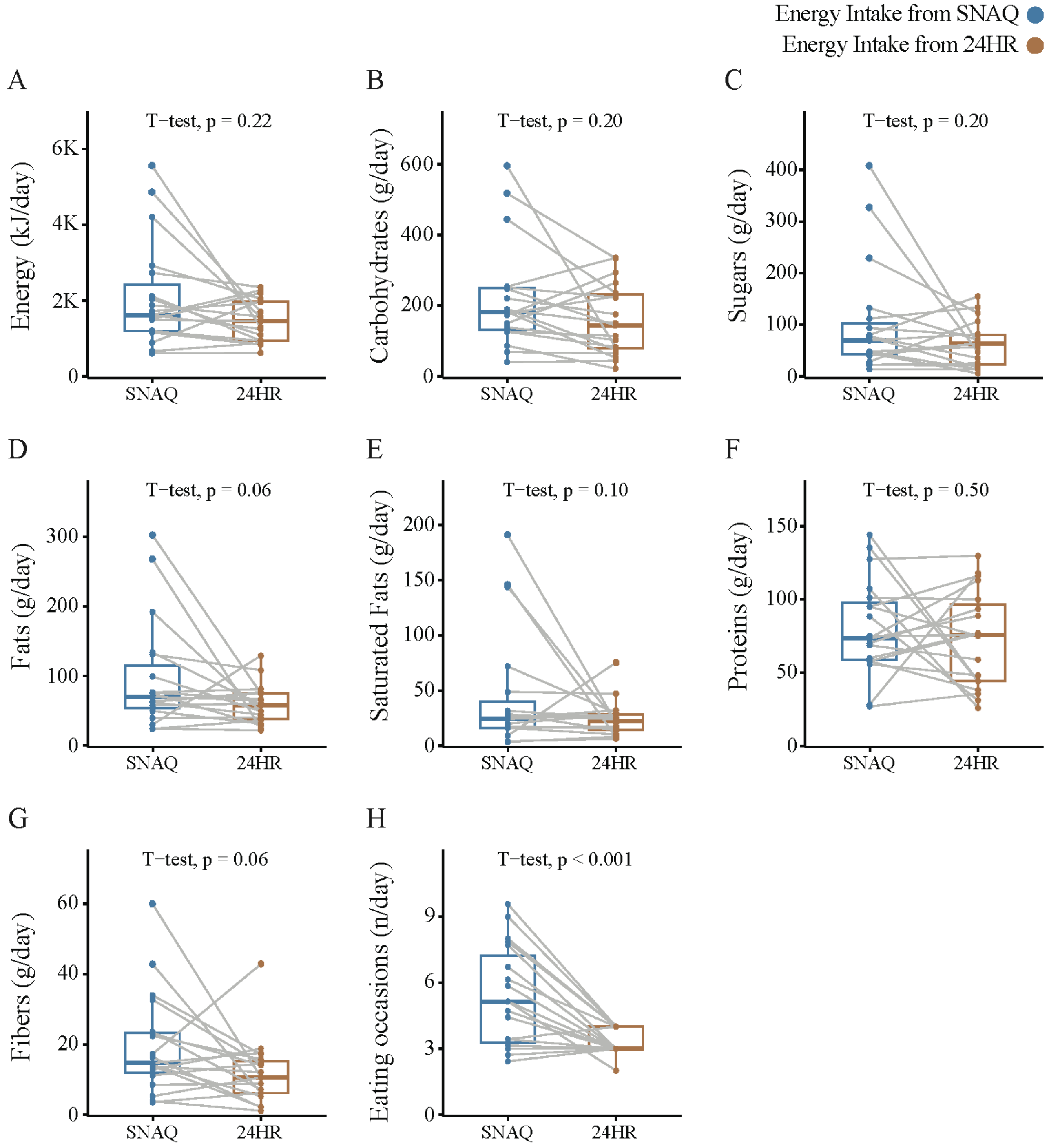

Supplementary Figure 6: Percentage of the measurement differences of total daily energy intake (A) macronutrient intake (B-G) and eating occasions (H) estimated with the SNAQ app in relation to the 24-hour dietary recall (24HR).

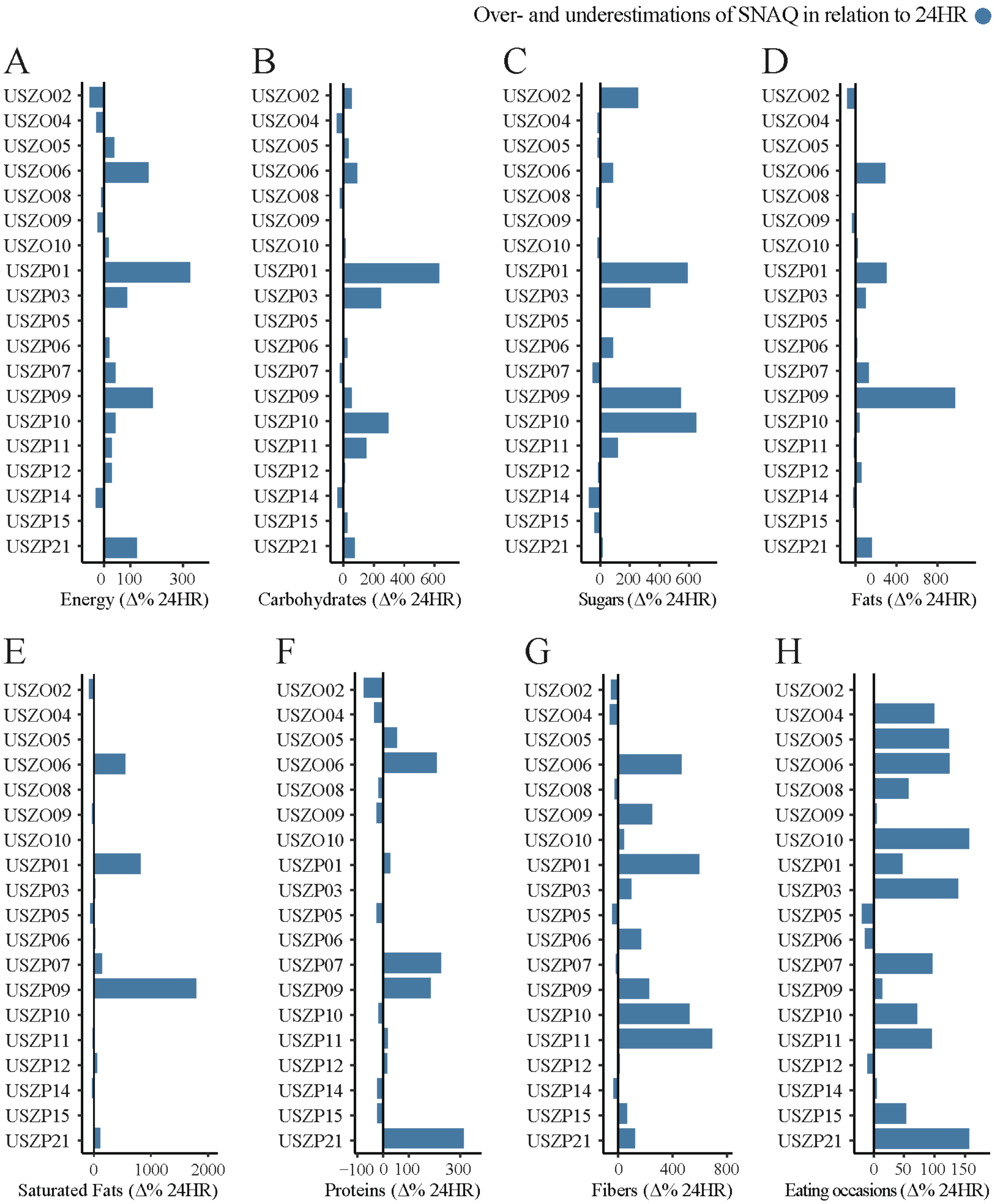

Supplementary Figure 7: Changes in body weight during the study period and correlation analysis of linear models of relationship between measurement differences of SNAQ and the 24-hour dietary recall (24HR).

(A) Changes of body weight of the study participants during the study week. Bars on the left of the y-axis represent body weight loss. Bars on the right of the y-axis represent body weight gain. (B) Linear model of the relationship between changes of body weight during the study week and percentage of measurement differences of daily energy intake estimated with SNAQ in relation to DLW. (C) Linear model of the relationship between estimations of daily energy expenditure during the study week and percentage of measurement differences of daily energy intake estimated with SNAQ in relation to DLW. (D) Linear model of the relationship between changes of body weight during the study week and percentage of measurement differences of daily energy intake estimated with 24HR in relation to DLW. (E) Linear model of the relationship between estimations of daily energy expenditure during the study week and percentage of measurement differences of daily energy intake estimated with 24HR in relation to DLW. Δ=difference from baseline. %Δ=percentage difference. *24HR*=24-hour dietary recall. *DLW*=doubly labelled water. *K*= decimal unit suffix for thousand. *P*=p-value of the coefficient of determination. R^2=coefficient of determination of the linear relationship.

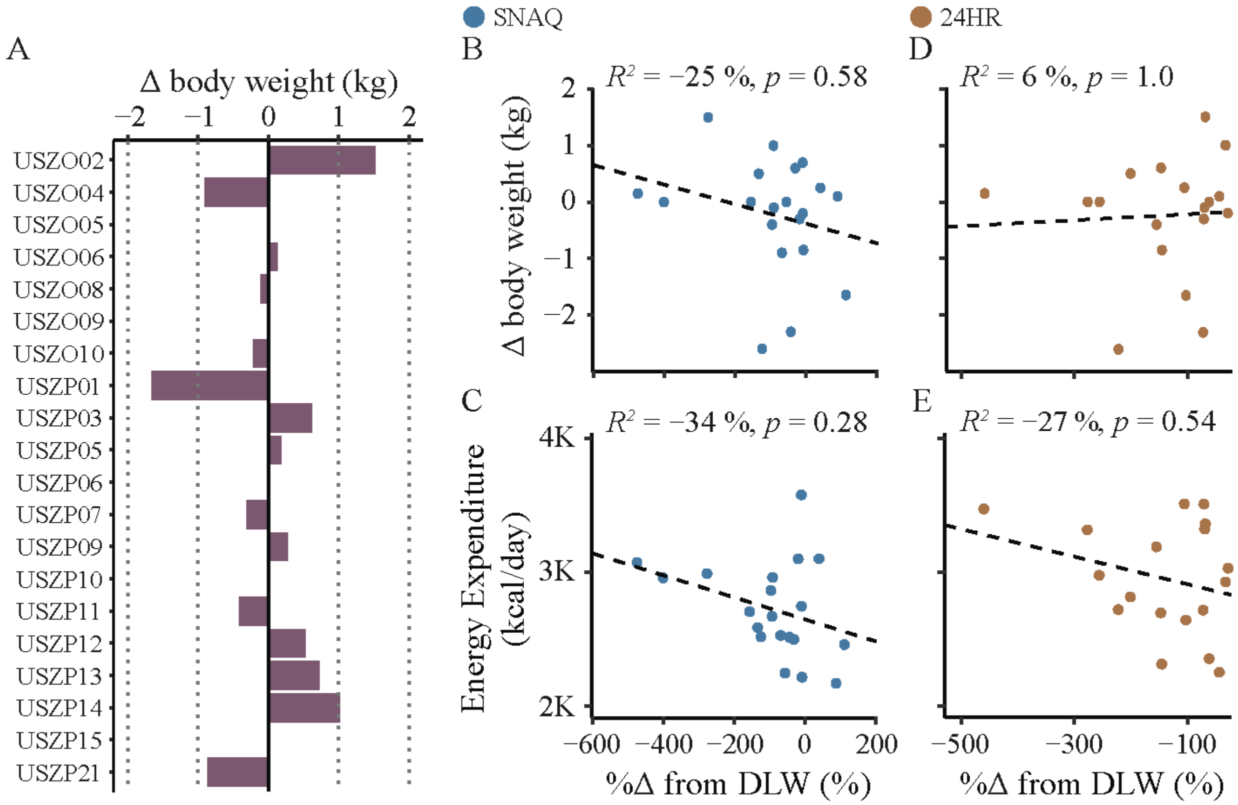
SNAQ
24HR
A
Δ body weight (kg)
−2
−1
0
1
2
USZO02
USZO04
USZO05
USZO06
USZO08
USZO09
USZO10
USZP01
USZP03
USZP05
USZP06
USZP07
USZP09
USZP10
USZP11
USZP12
USZP13
USZP14
USZP15
USZP21
B
R² = −25 %, p = 0.58
Δ body weight (kg)
D
R² = 6 %, p = 1.0
C
R² = −34 %, p = 0.28
Energy Expenditure (kcal/day)
4K
3K
2K
E
R² = −27 %, p = 0.54
−600
−400
−200
0
200
−500
−300
−100
%Δ from DLW (%)
%Δ from DLW (%)

Supplementary Figure 8: Linear model of the relationship between daily energy intake estimated with SNAQ and daily energy intake estimated with the 24-hour dietary recall (24HR)

24HR=24-hour dietary recall. *p*=p-value of the coefficient of determination. R^2=coefficient of determination of the linear model.

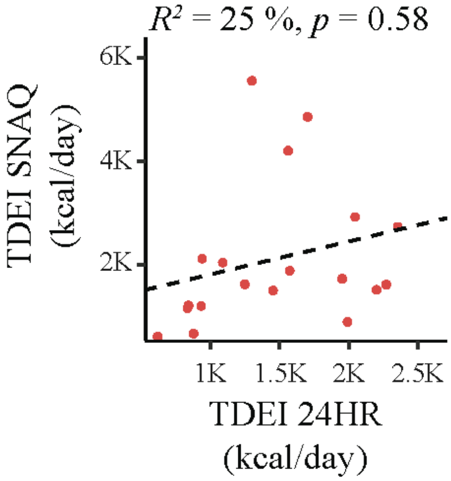

Supplementary tables

Supplementary Table 1: Reporting of the study according to the STROBE-nut checklist table.

Reporting checklist for observational studies in nutritional epidemiology.

Based on the STROBE-nut guidelines.

		Reporting Item	Page Number
**Title and abstract**			
Title	#1a	Indicate the study’s design with a commonly used term in the title or the abstract	1
Abstract	#1b	Provide in the abstract an informative and balanced summary of what was done and what was found	4
None	#nut-1	State the dietary/nutritional assessment method(s) used in the title or in the abstract.	1
**Introduction**			
Background / rationale	#2	Explain the scientific background and rationale for the investigation being reported	6
Objectives	#3	State specific objectives, including any prespecified hypotheses	7
**Methods**			
Study design	#4	Present key elements of study design early in the paper	8
Setting	#5	Describe the setting, locations, and relevant dates, including periods of recruitment, exposure, follow-up, and data collection	8
Eligibility	#6a	Cohort study: Give the eligibility criteria and the sources and methods of selection of participants. Describe methods of follow-up. Case-control study: Give the eligibility criteria and the sources and methods of case ascertainment and control selection. Give the rationale for the choice of cases and	8

		controls. Cross-sectional study: Give the eligibility criteria, and the sources and methods of selection of participants.	
None	#6b	Cohort study: For matched studies, give matching criteria and number of exposed and unexposed. Case-control study: For matched studies, give matching criteria and the number of controls per case.	n/a
Variables	#7	Clearly define all outcomes, exposures, predictors, potential confounders, and effect modifiers. Give diagnostic criteria, if applicable	9
Data sources and measurement	#8	For each variable of interest give sources of data and details of methods of assessment (measurement). Describe comparability of assessment methods if there is more than one group. Give information separately for for exposed and unexposed groups if applicable.	12
Bias	#9	Describe any efforts to address potential sources of bias	17
Study size	#10	Explain how the study size was arrived at	18
Quantitative variables	#11	Explain how quantitative variables were handled in the analyses. If applicable, describe which groupings were chosen, and why	19
Statistical methods	#12a	Describe all statistical methods, including those used to control for confounding	
19			
Subgroups and interactions	#12b	Describe any methods used to examine subgroups and interactions	n/a
Missing data	#12c	Explain how missing data were addressed	19
Loss to follow up	#12d	Cohort study: if applicable, explain how loss to follow-up was addressed. Case-control study: if applicable, explain how matching of cases and controls was addressed. Cross-sectional study: if applicable, describe analytical methods taking account of sampling strategy.	n/a
Sensitivity analysis	#12e	Describe any sensitivity analyses	

NA			
None	#nut-5	Describe any characteristics of the study settings that might affect the dietary intake or nutritional status of the participants, if applicable.	10
None	#nut-6	Report any particular dietary, physiologic, or nutritional characteristics that were considered when selecting the target population.	8
None	#nut-7.1	Clearly define foods, food groups, nutrients, or other food components (e.g., preparation method, taxonomical descriptors, classification, chemical form).	n/a
None	#nut-7.2	When calculating dietary patterns, describe the methods to obtain them and their nutritional properties.	9
None	#nut-8.1	Describe the dietary assessment method(s) (e.g., portion size estimation, number of days and items recorded, how it was developed and administered, and how quality was ensured); report if and how supplement intake was assessed.	15
None	#nut-8.2	Describe and justify food-composition data used; explain the procedure to match food composition with consumption data; describe the use of conversion factors, if applicable	15
None	#nut-8.3	Describe the nutrient requirements, recommendations, or dietary guidelines and the evaluation approach used to compare intake with the dietary reference values, if applicable	n/a
None	#nut-8.4	When using nutritional biomarkers, additionally use the STROBE-ME; report the type of biomarkers used and usefulness as dietary exposure markers	14
None	#nut-8.5	Describe the assessment of nondietary data (e.g., nutritional status and influencing factors) and timing of the assessment of these variables in relation to dietary assessment	n/a
None	#nut-8.6	Report on the validity of the dietary or nutritional assessment methods and any internal or external validation used in the study, if applicable	14
None	#nut-9	Report how bias in dietary or nutritional assessment was addressed (e.g., misreporting, changes in habits as a result of	17

		being measured, data imputation from other sources).	
None	#nut-11	Explain categorization of dietary/nutritional data (e.g., use of N-tiles and handling of nonconsumers) and the choice of reference category, if applicable.	19
None	#nut-12.1	Describe any statistical method used to combine dietary or nutritional data, if applicable.	19
None	#nut-12.2	Describe and justify the method for energy adjustments, intake modeling, and use of weighting factors, if applicable	n/a
None	#nut-12.3	Report any adjustments for measurement error (i.e., from a validity or calibration study).	n/a
**Results**			
Participants	#13a	Report numbers of individuals at each stage of study—eg numbers potentially eligible, examined for eligibility, confirmed eligible, included in the study, completing follow-up, and analysed. Give information separately for for exposed and unexposed groups if applicable.	22
Non-participation	#13b	Give reasons for non-participation at each stage	22
Participant journey	#13c	Consider the use of a flow diagram	
NA			
Descriptive data	#14a	Give characteristics of study participants (eg demographic, clinical, social) and information on exposures and potential confounders. Give information separately for exposed and unexposed groups if applicable.	22
Missing data	#14b	Indicate number of participants with missing data for each variable of interest	
22			
Follow-up time	#14c	Cohort study: Summarise follow-up time (eg, average and total amount)	
NA			
Outcome data	#15	Cohort study: report numbers of outcome events or summary	22

		measures over time. Case-control study: report numbers in each exposure category, or summary measures of exposure. Cross-sectional study: report numbers of outcome events or summary measures.	
Main results	#16a	Give unadjusted estimates and, if applicable, confounder-adjusted estimates and their precision (eg, 95% confidence interval). Make clear which confounders were adjusted for and why they were included	22
Category boundaries	#16b	Report category boundaries when continuous variables were categorized	22
Relative and absolute risks	#16c	If relevant, consider translating estimates of relative risk into absolute risk for a meaningful time period	
NA			
Other analyses	#17	Report other analyses done—eg analyses of subgroups and interactions, and sensitivity analyses	NA
None	#nut-13	Report the number of individuals excluded on the basis of missing, incomplete, or implausible dietary and nutritional data.	22
None	#nut-14	Give the distribution of participant characteristics across the exposure variables, if applicable; specify if food consumption for the total population or consumers only was used to obtain results	n/a
None	#nut-16	Specify if nutrient intakes are reported with or without the inclusion of dietary supplement intake, if applicable.	n/a
None	#nut-17	Report any sensitivity analysis (e.g., exclusion of misreporters or outliers) and data imputation, if applicable	25
**Discussion**			
Key results	#18	Summarise key results with reference to study objectives	29
Limitations	#19	Discuss limitations of the study, taking into account sources of potential bias or imprecision. Discuss both direction and magnitude of any potential bias.	35
Interpretation	#20	Give a cautious overall interpretation considering objectives,	30

		limitations, multiplicity of analyses, results from similar studies, and other relevant evidence.	
Generalisability	#21	Discuss the generalisability (external validity) of the study results	34
None	#nut-19	Describe the main limitations of the data sources and assessment methods used and implications for the interpretation of the findings	35
None	#nut-20	Report the nutritional relevance of the findings, given the complexity of diet or nutrition as an exposure.	34
**Other Information**			
Funding	#22	Give the source of funding and the role of the funders for the present study and, if applicable, for the original study on which the present article is based	2
Ethics	#nut-22.1	Describe the procedure for consent and study approval from ethics committee(s).	8
Data statement	#nut-22.2	Provide data collection tools and data as online material or explain how they can be accessed	37

The STROBE-nut checklist is distributed under the terms of the Creative Commons Attribution License CC-BY. This checklist was completed on 05. July 2023 using https://www.goodreports.org/, a tool made by the EQUATOR Network in collaboration with Penelope.ai

Supplementary Table 2: Eligibility criteria

**Inclusion Criteria**
• Adult aged 18 years or older<br>• BMI of 30 kg/m^2 or larger<br>• No history of bariatric surgery<br>• Independently mobile<br>• Digital literacy<br>• Ability to communicate fluently in English or German<br>• Capacity to consent to participate.
**Exclusion Criteria**
• Pregnancy/lactation<br>• History of have undergone any metabolic or bariatric surgery<br>• Inability to understand instructions<br>• Systemic or gastrointestinal condition which may affect food intake or preference<br>• Diabetes Mellitus (type I and II)<br>• Medically prescribed diets intended for weight loss or weight gain that intentionally constrain total energy intake or eating behaviour<br>• Active and significant psychiatric illness including substance misuse<br>• Suffering from heart or kidney failure or malabsorption<br>• Significant cognitive or communication issues<br>• Medications with documented effect on food intake or food preference<br>• History of significant food allergy and certain dietary restrictions<br>• Recent travel history (overnight trip of more than 200 miles) within 2 weeks before or during the study period after dose administration of the doubly labelled water<br>• Need for intravenous therapy during 2 weeks before and during the study period

Supplementary Table 3: Doses of doubly labelled water (DLW) for each study participant and relative DLW batch

Study identifier	DLW dose (mL)	DLW batch*
USZO02	101.1689	Batch 1
USZO04	100.6944	Batch 2
USZO05	101.3982	Batch 2
USZO06	101.1638	Batch 2
USZO08	111.3606	Batch 3
USZO09	101.044	Batch 2
USZO10	101.5941	Batch 2
USZP01	100.1023	Batch 1
USZP03	100.137	Batch 1
USZP04	100.0811	Batch 1
USZP05	100.7038	Batch 1
USZP06	101.3027	Batch 1
USZP07	100.264	Batch 2
USZP08	100.6855	Batch 2
USZP09	101.0574	Batch 2
USZP10	102.9755	Batch 2
USZP11	101.5197	Batch 2
USZP12	100.7739	Batch 2
USZP13	100.3823	Batch 1
USZP14	100.2059	Batch 2
USZP15	101.3051	Batch 2
USZP21	100.1933	Batch 3

* The description of the composition of the three doubly labelled water batches is available in supplementary table 5.

Supplementary Table 4: Description of the composition of the three doubly labelled water batches

DLW Batch	Batch date	Deuterium (g)	Lot Number D_2O	18-Oxygen (g)	Lot Number $H_2^{18}O$
Batch 1	06.05.2020	120.64	I-22828E	1803.23	I-E2581
Batch 2	28.01.2021	121.47	I-22828E	1802.69	I-E2581
Batch 3	16.08.2022	42.59	PR-33098	632.7	I-E2581

Supplementary Table 5: Calculations of BMR performed using both the actual and adjusted patient body weights according to the equations of both Mifflin-St Jeor and Harris-Benedict and estimates of Resting Energy Expenditure (REE) calculated with seca analytics 125 using a proprietary regression model based on impedance-derived body composition.

Study identifier	Height (m)	Body weight (kg)	Age (years)	IBW (kg)	ABW (kg)	BMR (Harris-Benedict)	BMR (Mifflin-St Jeor)	aBMR (Harris-Benedict)	aBMR (Mifflin-St Jeor)	REE (seca)
USZO02	1.75	103.9	25	70.5	80.5	1937.3	1846.3	1694.2	1612.6	NA
USZO04	1.63	108.8	64	59.6	74.3	1910.1	1625.3	1552.3	1281.2	1723
USZO05	1.63	97.6	42	59.6	71.0	1794.2	1623.8	1517.5	1357.7	1585
USZO06	1.64	82.9	39	60.5	67.2	1647.8	1498.0	1484.8	1341.2	1540
USZO08	1.68	102.5	34	64.1	75.6	1877.7	1744.0	1598.3	1475.4	1681
USZO09	1.54	87.5	38	51.4	62.3	1630.5	1486.5	1368.1	1234.1	1558
USZO10	1.72	103.0	52	67.7	78.3	1908.9	1684.0	1652.3	1437.2	1682
USZP01	1.58	102.9	40	55.1	69.4	1816.7	1655.5	1468.5	1320.7	1668
USZP03	1.71	112.1	36	66.8	80.4	1997.1	1848.8	1667.6	1531.9	1776
USZP04	1.66	114.5	18	62.3	78.0	1988.9	1931.0	1609.4	1566.1	NA
USZP05	1.66	99.9	30	62.3	73.6	1837.6	1725.5	1564.0	1462.4	2191
USZP06	1.66	99.2	19	62.3	73.4	1830.3	1773.5	1561.8	1515.3	1646
USZP07	1.68	101.2	27	64.1	75.2	1863.6	1765.5	1594.1	1506.3	1945
USZP08	1.69	158.5	34	65.0	93.1	2466.6	2310.3	1786.1	1656.0	NA
USZP09	1.58	105.8	45	55.1	70.3	1846.9	1659.5	1477.6	1304.4	1978
USZP10	1.70	126.8	63	65.9	84.2	2143.4	1854.5	1700.3	1428.5	2129
USZP11	1.74	105.8	55	69.6	80.4	1950.5	1709.0	1687.1	1455.7	1695
USZP12	1.69	131.3	31	65.0	84.9	2183.2	2052.8	1701.1	1589.2	1876
USZP13	1.68	116.9	45	64.1	79.9	2026.9	1832.5	1643.1	1463.4	1702
USZP14	1.60	107.0	21	56.9	71.9	1871.9	1803.5	1507.4	1453.0	1922
USZP15	1.75	103.9	25	70.5	80.5	1937.3	1846.3	1694.2	1612.6	NA
USZP21	1.63	108.8	64	59.6	74.3	1910.1	1625.3	1552.3	1281.2	2070

aBMR=adjusted basal metabolic rate. ABW=adjusted body weight. BMR=basal metabolic rate. IBW=ideal body weight.

Supplementary Table 6: Baseline bioelectrical impedance vector analysis of the study population

	Cohort – Obesity, N = 20*	
	**Bioelectrical impedance**	**Isotope Dilution**
**Fat mass**		
Fat mass (kg)	52.9 (12.5)	54.4 (17.3)
Fat mass (%)	48.6 (4.2)	49.3 (8.8)
Fat mass index (kg/m^2)	19.4 (4.3)	*n/a*
**Fat-free mass**		
Fat-free mass (kg)	54.9 (6.3)	54.0 (6.0)
Fat-free mass (%)	51.3 (4.2)	*n/a*
Fat-free mass index (kg/m^2)	20.0 (1.9)	*n/a*
**Body water**		
Total body water (L)	41.2 (4.9)	39.5 (4.4)
Total body water (%)	38.3 (3.1)	*n/a*
Extracellular water (L)	18.5 (2.1)	*n/a*
Extracellular water (%)	17.2 (1.2)	*n/a*
ECW/TBW	44.9 (1.5)	*n/a*
**Visceral fat (L)**	4.3 (2.4)	

* Mean (SD). SD, standard deviation.
ECW=extracellular water. *n/a*=not available. *TBW*=total body water.

Supplementary Table 7: Nationality of the study population

	Cohort
	**Obesity, N = 20***
**Nationality**	
Algeria	1 / 20 (5.0 %)
China	1 / 20 (5.0 %)
France	1 / 20 (5.0 %)
Germany	3 / 20 (15.0 %)
India	1 / 20 (5.0 %)
Italy	2 / 20 (10.0 %)
Kosovo	1 / 20 (5.0 %)
Serbia	1 / 20 (5.0 %)
Spain	2 / 20 (5.0 %)
Switzerland	6 / 20 (30.0 %)

* Frequency (%). %=percentage.

Supplementary Table 8: Results of the doubly labelled water (DLW) analysis

Study identifier	TBW_d (L)	TBW_O (L)	TBW (L)	TBW (mol)	rCO_2 (ppm)	TDEE (kcal/day)	PAL	AEE (kcal/day)	Turnover (L)	%TBF
USZO02	41.4	41.1	41.3	2291.2	586.0	3364.5	2.09	1415.5	−3.9	45.7
USZO04	40.0	40.2	40.1	2225.6	477.1	2739.1	2.14	1184.0	−3.7	49.6
USZO05	39.8	40.0	39.9	2214.9	519.1	2980.1	2.19	1324.3	−3.5	44.1
USZO06	31.3	31.3	31.3	1736.2	392.9	2255.6	1.68	688.8	−2.2	48.5
USZO08	39.3	39.3	39.3	2183.1	579.1	3324.9	2.25	1517.0	−4.2	47.6
USZO09	42.4	42.3	42.3	2349.2	578.3	3320.1	2.69	1754.0	−5.4	33.9
USZO10	38.1	37.4	37.8	2096.2	528.3	3032.8	2.11	1292.3	−3.9	49.9
USZP01	41.2	40.9	41.0	2276.7	460.9	2646.2	2.00	1060.9	−3.3	45.5
USZP03	35.5	35.2	35.3	1961.3	470.2	2699.4	1.76	897.5	−3.2	56.9
USZP05	39.5	39.2	39.3	2184.2	605.5	3476.4	2.22	1562.7	−2.6	53.0
USZP06	39.0	38.8	38.9	2158.9	473.8	2720.0	1.86	985.6	−3.1	46.8
USZP07	41.0	40.5	40.7	2261.8	611.7	3512.2	2.32	1645.6	−2.8	43.9
USZP09	51.0	50.7	50.8	2822.2	612.0	3513.9	2.33	1656.1	−3.1	31.3
USZP10	41.1	41.3	41.2	2286.5	474.4	2723.8	1.64	795.5	−3.5	64.5
USZP11	43.2	42.7	42.9	2383.8	556.1	3192.6	2.45	1568.9	−4.2	44.5
USZP12	35.5	35.3	35.4	1964.1	490.9	2818.2	1.97	1107.9	−4.1	61.9
USZP13	44.5	44.0	44.3	2456.9	724.5	4159.5	2.86	2287.9	−2.5	42.8
USZP14	41.6	41.3	41.5	2301.4	510.5	2931.2	1.84	1048.9	−2.0	56.8
USZP15	31.5	31.6	31.5	1750.1	410.6	2357.4	1.61	658.3	−3.6	63.1
USZP21	35.6	35.1	35.4	1962.5	403.5	2316.9	1.59	632.2	−3.2	54.8

TBW_d=total body water estimated with deuterium. TBW_O=total body water estimated with oxygen18. TBW=average estimation of total body water. rCO_2=daily respiratory carbon dioxide. TDEE=total daily energy expenditure. PAL=physical activity level. AEE=activity-related energy expenditure. Turnover=water turnover during the study week. %TBF=percentage of the total body fat.

Supplementary Table 9: Dilution spaces of the isotopes deuterium and 18-oxygen for each study participant

Study identifier	N_d	N_O	N_d/N_O
USZO02	43.13824616	41.40096908	1.041962232
USZO04	41.66439527	40.44742301	1.030087758
USZO05	41.47110015	40.24539115	1.030455885
USZO06	32.55702182	31.50003241	1.033555185
USZO08	40.95265406	39.59291288	1.034343045
USZO09	44.09198506	42.58392624	1.035413804
USZO10	39.66851194	37.68237622	1.052707284
USZP01	42.85308376	41.15157221	1.041347425
USZP03	36.95401366	35.4137313	1.04349393
USZP05	41.16855611	39.4260336	1.044197256
USZP06	40.56969795	39.08602878	1.037959067
USZP07	42.69923417	40.76023043	1.047570971
USZP09	53.10884321	51.02183992	1.040904117
USZP10	42.79887576	41.55987609	1.029812401
USZP11	44.96895216	42.99095894	1.046009516
USZP12	36.95379277	35.51661342	1.040464988
USZP13	46.35350069	44.30190573	1.046309407
USZP14	43.27743491	41.6382099	1.039368287
USZP15	32.78974649	31.77855613	1.031819896
USZP21	37.05484747	35.36166885	1.047881751

N_d=dilution space of the isotope deuterium (^2H). N_O=dilution space of the isotope oxygen18 (^{18}O). N_d/N_O=dilution space ratio.

Supplementary Table 10: Elimination rates of the isotopes deuterium and 18-oxygen for each study participant

Study identifier	k_d	k_O	k_d/k_O
USZO02	−0.084062165	−0.112123206	1.333812979
USZO04	−0.096580742	−0.121032814	1.253177518
USZO05	−0.082343407	−0.108244411	1.314548604
USZO06	−0.086884569	−0.112157577	1.290880279
USZO08	−0.084703535	−0.113718492	1.342547181
USZO09	−0.092211126	−0.119622133	1.297263551
USZO10	−0.080697494	−0.108269221	1.341667692
USZP01	−0.110209425	−0.133981069	1.215695195
USZP03	−0.083913497	−0.110397047	1.315605367
USZP05	−0.063047884	−0.092458182	1.466475562
USZP06	−0.069258716	−0.09323147	1.346133377
USZP07	−0.086896437	−0.116497814	1.340651212
USZP09	−0.075669581	−0.099619141	1.316501831
USZP10	−0.077023241	−0.100102722	1.299643083
USZP11	−0.069273482	−0.094601236	1.365619757
USZP12	−0.100252600	−0.128318965	1.279956483
USZP13	−0.070698923	−0.102113657	1.444345286
USZP14	−0.085002574	−0.109772985	1.291407762
USZP15	−0.114548082	−0.141627237	1.2363999
USZP21	−0.070272373	−0.092927652	1.322392403

k_d=elimination rate of the isotope deuterium (^2H). k_O=elimination rate of the isotope oxygen18 (^{18}O). k_d/k_O=elimination rate ratio.

Supplementary Table 11: Results of the Shapiro–Wilk test for normality of the distribution of the energy estimates of the SNAQ app, the 24-hour dietary recall, and the DLW technique

	**Statistic***	**p-value***
SNAQ	0.87	0.01
DLW	0.93	0.51
24HR	0.96	0.21

* Shapiro–Wilk test.
DLW=doubly labelled water technique. *SNAQ*=the SNAQ app. *24HR*=24-hour dietary recall.

Supplementary Table 12: Absolute and percentage measurement differences of total daily energy intake estimated with the SNAQ app in relation to total daily energy expenditure estimated with DLW technique

Study identifier	Absolute difference (kcal/day)	Percentage difference (%)
USZO02	−2473.4	−73.5
USZO04	−1126.5	−41.1
USZO05	−1822.1	−61.1
USZO06	1951.7	86.5
USZO08	−1599.5	−48.1
USZO09	−2658.6	−80.1
USZO10	−302.1	−10.0
USZP01	2916.1	110.2
USZP03	−660.5	−24.5
USZP05	−2872.0	−82.6
USZP06	−840.7	−30.9
USZP07	−589.3	−16.8
USZP09	1349.0	38.4
USZP10	−1515.1	−55.6
USZP11	−1575.0	−49.3
USZP12	−1618.5	−57.4
USZP13	−426.4	−10.3
USZP14	−1419.2	−48.4
USZP15	−861.0	−36.5
USZP21	−201.6	−8.7

Supplementary Table 13: Absolute and percentage measurement differences of total daily energy intake estimated with the 24-hour dietary recall in relation to estimates with the DWL technique

Study identifier	Absolute difference (kcal/day)	Percentage difference (%)
USZO02	−1372.7	−40.8
USZO04	−466.9	−17.0
USZO05	−2144.2	−72.0
USZO06	−692.3	−30.7
USZO08	−1371.1	−41.2
USZO09	−2440.8	−73.5
USZO10	−678.3	−22.4
USZP01	−1344.6	−50.8
USZP03	−1608.9	−59.6
USZP05	−2855.8	−82.2
USZP06	−1145.6	−42.1
USZP07	−1465.0	−41.7
USZP09	−1809.3	−51.5
USZP10	−1880.3	−69.0
USZP11	−1942.3	−60.8
USZP12	−1883.8	−66.8
USZP14	−729.8	−24.9
USZP15	−903.7	−38.3
USZP21	−1374.9	−59.3

Supplementary Table 14: Comparison of energy intake, macronutrient intake, and eating occasions between SNAQ and 24HR

	Dietary assessment method		Difference[†]	95 % CI[†,‡]	p-value[†]
	SNAQ, N = 20*	24HR, N = 20*			
**Energy intake****					
Energy (kcal/day)	2,105.6 (1,389.8)	1,464.0 (554.1)	641.7 (+44 %)	–67, 1,351	0.22
Energy (kJ/day)	8,810.0 (5,815.0)	6,125.2 (2,318.3)	2,684.8 (+44 %)	–282, 5,652	0.22
**Macronutrient intake**					
Carbohydrates (g/day)	220.0 (147.6)	159.1 (100.5)	60.8 (+38 %)	–23, 144	0.2
Sugars (g/day)	101.0 (107.1)	62.1 (43.5)	38.9 (+63 %)	–16, 94	0.2
Fats (g/day)	96.7 (78.4)	59.1 (28.1)	37.7 (+64 %)	–12.0, 19.0	0.06
Saturated Fats (g/day)	45.7 (54.1)	24.1 (16.0)	21.6 (+90 %)	–5, 48	0.1
Proteins (g/day)	81.2 (32.4)	73.2 (32.7)	8.0 (+11 %)	–13, 29	0.5
Fibers (g/day)	19.7 (14.3)	12.0 (9.3)	7.7 (+64 %)	–0.3, 16	0.06
**Temporal organization**					
Eating occasions (n/day)	5.4 (2.3)	3.3 (0.6)	2.1 (+63 %)	–1.0, 3.2	< 0.001

* Mean (SD). SD, standard deviation.
** Total daily energy intake.
† Welch Two Sample t-test.
‡ CI = Confidence interval

Supplementary Table 15: Overall difference, and over- and underestimations of total daily energy intake estimated with SNAQ and 24HR in relation to DLW

		n	Δ DLW, N = 20*	MPD DLW, N = 20*	$p^{\dagger}$
Overall difference	**Energy intake SNAQ**<br>Energy (kcal/day)<br>Energy (kJ/day)	30	−817.2 (1474.5)<br>−3,420.1 (6,172.6)	−25.0 % (51.1)	0.01
	**Energy intake 24HR**<br>Energy (kcal/day)<br>Energy (kJ/day)	30	−1,479.5 (635.5)<br>−6,193.8 (2,659.2)	−49.7 % (18.7)	<0.001
Overestimations	**Energy intake SNAQ**<br>Energy (kcal/day)<br>Energy (kJ/day)	4	2,072.3 (790.5)<br>8,670.3 (3,308.2)	78.4 % (36.6)	
	**Energy intake 24HR**<br>Energy (kcal/day)<br>Energy (kJ/day)	0	n/a<br>n/a	n/a	
Underestimations	**Energy intake SNAQ**<br>Energy (kcal/day)<br>Energy (kJ/day)	26	−1,327.1 (813.7)<br>−5,555.6 (3,403.7)	−43.2 % (24.0)	
	**Energy intake 24HR**<br>Energy (kcal/day)<br>Energy (kJ/day)	20	−1,479 (635.5)<br>−6,190.6 (2,659.2)	−49.7 % (18.7)	

* Mean (SD). SD, standard deviation.

† Wilcoxon signed-rank test

24HR=24-hour dietary recall. DLW=doubly labelled water. MPD DLW=mean percentage difference from DLW values. n=number of cases. NA=not available. Δ DLW=difference from DLW values.

Supplementary Table 16: Over- and underestimations of energy and macronutrient intake estimated with SNAQ in relation to 24HR

		n	Δ 24HR, N = 30*	Δ% 24HR, N = 30*
**Overestimations**	**Energy intake****			
	Energy (kcal/day)	*14*	1,161.9 (1,332.03)	+85.7 % (93.6)
	Energy (kJ/day)	*14*	4,861.2 (5,573.2)	+85.7 % (93.6)
	**Macronutrient intake**			
	Carbohydrates (g/day)	*15*	108.9 (132.3)	+121.8 % (171.2)
	Sugars (g/day)	*11*	98.1 (117.3)	+268.7 % (246.2)
	Fats (g/day)	*13*	71.8 (85.8)	+175.7 % (270.4)
	Saturated Fats (g/day)	*11*	50.8 (66.0)	+352.9 % (574.8)
	Proteins (g/day)	*10*	43.1 (39.4)	+115.7 % (116.6)
	Fibers (g/day)	*13*	16.0 (13.9)	+271.9 % (234)
	**Temporal organization**			
	Dietary occasions (n/day)	*17*	2.7 (1.8)	+83.1 % (52.1)
**Underestimations**	**Energy intake**			
	Energy (kcal/day)	*6*	−485.3 (402.1)	−25.8 % (18.1)
	Energy (kJ/day)	*6*	−2,030.8 (1,682.5)	−25.8 % (18.1)
	**Macronutrient intake**			
	Carbohydrates (g/day)	*5*	−73.8 (48.3)	−27.7 % (14.4)
	Sugars (g/day)	*9*	−26.8 (29.3)	−27.6 % (22.3)
	Fats (g/day)	*7*	−20.8 (35.1)	−22.5 % (26.2)
	Saturated Fats (g/day)	*9*	−10.9 (21.0)	−30.3 % (27.9)
	Proteins (g/day)	*10*	−23.7 (25.8)	−24.5 % (20.9)
	Fibers (g/day)	*7*	−6.6 (8.6)	−32.4 % (19.8)
	**Temporal organization**			
	Dietary occasions (n/day)	*3*	−0.4 (0.3)	−10.7 % (8.1)

* Mean (SD). SD, standard deviation.
** Total daily energy intake.
Δ 24HR=difference from 24HR values. Δ% 24HR=percentage difference from 24HR values. n=number of over- or underestimations per variable

Supplementary Table 17: Classification of study participants as plausible, over- or under-reporters of total daily energy intake estimated with SNAQ according to the Goldberg cut-off method,[1] using adjusted Basal Metabolic Rate (BMR) estimated by the Mifflin-St Jeor equation and Physical Activity Level (PAL) calculated as a ratio of DLW-based estimates of total daily energy expenditures (TDEE) and BMR.

**Study identifier**	**Energy Intake (kcal/day)**	**BMR (kcal/day)**	**PAL (TDEE:BMR)**	**TDEI:BMR**	**Classification (I)***	**Classification (II)§**
USZO02	891.1	1612.6	2.09	0.55	under-reporter	under-reporter
USZO04	1612.6	1281.2	2.14	1.26	under-reporter	under-reporter
USZO05	1158.0	1357.7	2.19	0.85	under-reporter	under-reporter
USZO06	4207.3	1341.2	1.68	3.14	over-reporter	over-reporter
USZO08	1725.4	1475.4	2.25	1.17	under-reporter	under-reporter
USZO09	661.6	1234.1	2.69	0.54	under-reporter	under-reporter
USZO10	2730.7	1437.2	2.11	1.90	*plausible reporter*	under-reporter
USZP01	5562.3	1320.7	2.00	4.21	over-reporter	over-reporter
USZP03	2038.9	1531.9	1.76	1.33	under-reporter	under-reporter
USZP05	604.4	1566.1	2.22	0.39	under-reporter	under-reporter
USZP06	1879.3	1462.4	1.86	1.29	under-reporter	under-reporter
USZP07	2922.9	1515.3	2.32	1.93	*plausible reporter*	*plausible reporter*
USZP09	4862.9	1506.3	2.33	3.23	over-reporter	over-reporter
USZP10	1208.7	1656.0	1.64	0.73	under-reporter	under-reporter
USZP11	1617.6	1304.4	2.45	1.24	under-reporter	under-reporter
USZP12	1199.7	1428.5	1.97	0.84	under-reporter	under-reporter
USZP13	3733.1	1455.7	2.86	2.56	*plausible reporter*	over-reporter
USZP14	1512.0	1589.2	1.84	0.95	under-reporter	under-reporter
USZP15	1496.4	1463.4	1.61	1.02	under-reporter	under-reporter
USZP21	2115.3	1453.0	1.59	1.46	under-reporter	under-reporter

* Classification with an S factor specific for the study population.
§ Classification with an S factor according to Black .[1]
BMR=adjusted basal metabolic rate estimated by the Mifflin-St Jeor equation. *PAL*=physical activity level. *TDEE*=total daily energy expenditure. *TDEI*=total daily energy intake.

Supplementary Table 18: Classification of the study participants as plausible, over- or under-reporters of total daily energy intake estimated with the 24-hour dietary recall (24HR) according to the Goldberg cut-off method,[1] using adjusted Basal Metabolic Rate (BMR) estimated by the Mifflin-St Jeor equation and Physical Activity Level (PAL) calculated as a ratio of DLW-based estimates of total daily energy expenditures (TDEE) and BMR.

**Study identifier**	**Energy Intake (kcal/day)**	**BMR (kcal/day)**	**PAL (TDEE:BMR)**	**TDEI:BMR**	**Classification (I)***	**Classification (II)§**
USZO02	1991.9	1612.6	2.09	1.24	under-reporter	under-reporter
USZO04	2272.3	1281.2	2.14	1.77	*plausible reporter*	under-reporter
USZO05	835.9	1357.7	2.19	0.62	under-reporter	under-reporter
USZO06	1563.3	1341.2	1.68	1.17	under-reporter	under-reporter
USZO08	1953.8	1475.4	2.25	1.32	under-reporter	under-reporter
USZO09	879.4	1234.1	2.69	0.71	under-reporter	under-reporter
USZO10	2354.6	1437.2	2.11	1.64	under-reporter	under-reporter
USZP01	1301.7	1320.7	2.00	0.99	under-reporter	under-reporter
USZP03	1090.5	1531.9	1.76	0.71	under-reporter	under-reporter
USZP05	620.6	1566.1	2.22	0.40	under-reporter	under-reporter
USZP06	1574.4	1462.4	1.86	1.08	under-reporter	under-reporter
USZP07	2047.1	1515.3	2.32	1.35	under-reporter	under-reporter
USZP09	1704.6	1506.3	2.33	1.13	under-reporter	under-reporter
USZP10	843.5	1656.0	1.64	0.51	under-reporter	under-reporter
USZP11	1250.2	1304.4	2.45	0.96	under-reporter	under-reporter
USZP12	934.5	1428.5	1.97	0.65	under-reporter	under-reporter
USZP13	24HR not available					
USZP14	2201.4	1589.2	1.84	1.39	under-reporter	under-reporter
USZP15	1453.7	1463.4	1.61	0.99	under-reporter	under-reporter
USZP21	941.9	1453.0	1.59	0.65	under-reporter	under-reporter
USZO02	1991.9	1612.6	2.09	1.24	under-reporter	under-reporter

* Classification with an S factor specific for the study population.
§ Classification with an S factor according to Black .[1]
BMR=adjusted basal metabolic rate. *PAL*=physical activity level. *TDEE*=total daily energy expenditure. *TDEI*= total daily energy intake.

Supplementary Table 19: Classification of study participants as plausible, over- or under-reporters of total daily energy intake estimated with SNAQ according to the Goldberg cut-off method,[1] using adjusted Basal Metabolic Rate (BMR) estimated by the Mifflin-St Jeor equation and Physical Activity Level (PAL) selected based on lifestyle using the software seca analytics 125.

Study identifier	Energy Intake (kcal/day)	BMR (kcal/day)	PAL (seca)	TDEI:BMR	Classification (I)*	Classification (II)§
USZO02	PAL not available					
USZO04	1612.6	1281.2	1.6	1.26	*plausible reporter*	under-reporter
USZO05	1158.0	1357.7	1.6	0.85	under-reporter	under-reporter
USZO06	4207.3	1341.2	1.6	3.14	over-reporter	over-reporter
USZO08	1725.4	1475.4	1.8	1.17	under-reporter	under-reporter
USZO09	661.6	1234.1	1.8	0.54	under-reporter	under-reporter
USZO10	2730.7	1437.2	1.0	1.90	*plausible reporter*	over-reporter
USZP01	5562.3	1320.7	1.6	4.21	over-reporter	over-reporter
USZP03	2038.9	1531.9	1.6	1.33	*plausible reporter*	under-reporter
USZP05	604.4	1566.1	1.8	0.39	under-reporter	under-reporter
USZP06	1879.3	1462.4	1.8	1.29	*plausible reporter*	under-reporter
USZP07	2922.9	1515.3	1.4	1.93	*plausible reporter*	over-reporter
USZP09	4862.9	1506.3	1.6	3.23	over-reporter	over-reporter
USZP10	1208.7	1656.0	1.8	0.73	under-reporter	under-reporter
USZP11	1617.6	1304.4	1.4	1.24	*plausible reporter*	under-reporter
USZP12	1199.7	1428.5	1.8	0.84	under-reporter	under-reporter
USZP13	3733.1	1455.7	1.6	2.56	over-reporter	over-reporter
USZP14	1512.0	1589.2	1.4	0.95	under-reporter	under-reporter
USZP15	PAL not available					
USZP21	2115.3	1453.0	1.6	1.46	*plausible reporter*	under-reporter

* Classification with an S factor specific for the study population.
§ Classification with an S factor according to Black .[1]
BMR=adjusted basal metabolic rate. *PAL*=physical activity level. *TDEE*=total daily energy expenditure. *TDEI*=total daily energy intake.

Supplementary Table 20: Classification of study participants as plausible, over- or under-reporters of total daily energy intake estimated with the 24-hour dietary recall (24HR) according to the Goldberg cut-off method,[1] using adjusted Basal Metabolic Rate (BMR) estimated by the Mifflin-St Jeor equation and Physical Activity Level (PAL) selected based on lifestyle using the software seca analytics 125.

Study identifier	Energy Intake (kcal/day)	BMR (kcal/day)	PAL (seca)	TDEI:BMR	Classification (I)[*]	Classification (II)[§]
USZO02	PAL not available					
USZO04	2272.3	1281.2	1.6	1.77	*plausible reporter*	over-reporter
USZO05	835.9	1357.7	1.6	0.62	under-reporter	under-reporter
USZO06	1563.3	1341.2	1.6	1.17	under-reporter	under-reporter
USZO08	1953.8	1475.4	1.8	1.32	*plausible reporter*	under-reporter
USZO09	879.4	1234.1	1.8	0.71	under-reporter	under-reporter
USZO10	2354.6	1437.2	1.0	1.64	*plausible reporter*	*plausible reporter*
USZP01	1301.7	1320.7	1.6	0.99	under-reporter	under-reporter
USZP03	1090.5	1531.9	1.6	0.71	under-reporter	under-reporter
USZP05	620.6	1566.1	1.8	0.40	under-reporter	under-reporter
USZP06	1574.4	1462.4	1.8	1.08	under-reporter	under-reporter
USZP07	2047.1	1515.3	1.4	1.35	*plausible reporter*	under-reporter
USZP09	1704.6	1506.3	1.6	1.13	under-reporter	under-reporter
USZP10	843.5	1656.0	1.8	0.51	under-reporter	under-reporter
USZP11	1250.2	1304.4	1.4	0.96	under-reporter	under-reporter
USZP12	934.5	1428.5	1.8	0.65	under-reporter	under-reporter
USZP13	24HR not available					
USZP14	2201.4	1589.2	1.4	1.39	*plausible reporter*	under-reporter
USZP15	PAL not available					
USZP21	941.9	1453.0	1.6	0.65	under-reporter	under-reporter

* Classification with an S factor specific for the study population.
§ Classification with an S factor according to Black .[1]
BMR=adjusted basal metabolic rate. *PAL*=physical activity level. *TDEE*=total daily energy expenditure. *TDEI*=total daily energy intake.

Supplementary Table 21: Classification of study participants as plausible, over- or under-reporters of total daily energy intake estimated with SNAQ according to the Goldberg cut-off method,[1] using adjusted Basal Metabolic Rate (BMR) estimated by the Mifflin-St Jeor equation and Physical Activity Level (PAL) calculated as a ratio of DLW-based estimates of total daily energy expenditures (TDEE) and Resting Energy Expenditure (REE) calculated with seca analytics 125 using a proprietary regression model based on impedance-derived body composition.

Study identifier	Energy Intake (kcal/day)	BMR (kcal/day)	PAL (TDEE:REE)	TDEI:BMR	Classification (I)*	Classification (II)§
USZO02	PAL not available					
USZO04	1612.6	1281.2	1.59	1.26	*plausible reporter*	under-reporter
USZO05	1158.0	1357.7	1.88	0.85	under-reporter	under-reporter
USZO06	4207.3	1341.2	1.46	3.14	over-reporter	over-reporter
USZO08	1725.4	1475.4	1.98	1.17	under-reporter	under-reporter
USZO09	661.6	1234.1	2.13	0.54	under-reporter	under-reporter
USZO10	2730.7	1437.2	1.80	1.90	*plausible reporter*	over-reporter
USZP01	5562.3	1320.7	1.59	4.21	over-reporter	over-reporter
USZP03	2038.9	1531.9	1.52	1.33	*plausible reporter*	under-reporter
USZP05	604.4	1566.1	1.59	0.39	under-reporter	under-reporter
USZP06	1879.3	1462.4	1.65	1.29	*plausible reporter*	under-reporter
USZP07	2922.9	1515.3	1.81	1.93	*plausible reporter*	over-reporter
USZP09	4862.9	1506.3	1.78	3.23	over-reporter	over-reporter
USZP10	1208.7	1656.0	1.28	0.73	under-reporter	under-reporter
USZP11	1617.6	1304.4	1.88	1.24	under-reporter	under-reporter
USZP12	1199.7	1428.5	1.50	0.84	under-reporter	under-reporter
USZP13	3733.1	1455.7	2.44	2.56	over-reporter	over-reporter
USZP14	1512.0	1589.2	1.53	0.95	under-reporter	under-reporter
USZP15	PAL not available					
USZP21	2115.3	1453.0	1.12	1.46	*plausible reporter*	under-reporter

* Classification with an S factor specific for the study population.
§ Classification with an S factor according to Black .[1]
BMR= adjusted basal metabolic rate. *PAL*=physical activity level. *REE*=resting energy expenditure by seca analytics 125. *TDEE*=total daily energy expenditure. *TDEI*=total daily energy intake.

Supplementary Table 22: Classification of study participants as plausible, over- or under-reporters of total daily energy intake estimated with the 24-hour dietary recall (24HR) according to the Goldberg cut-off method,[1] using adjusted Basal Metabolic Rate (BMR) estimated by the Mifflin-St Jeor equation and Physical Activity Level (PAL) calculated as a ratio of DLW-based estimates of total daily energy expenditures (TDEE) and Resting Energy Expenditure (REE) calculated with seca analytics 125 using a proprietary regression model based on impedance-derived body composition.

Study identifier	Energy Intake (kcal/day)	BMR (kcal/day)	PAL (TDEE:REE)	TDEI:BMR	Classification (I)*	Classification (II)§
USZO02	PAL not available					
USZO04	2272.3	1281.2	1.59	1.77	*plausible reporter*	*plausible reporter*
USZO05	835.9	1357.7	1.88	0.62	under-reporter	under-reporter
USZO06	1563.3	1341.2	1.46	1.17	under-reporter	under-reporter
USZO08	1953.8	1475.4	1.98	1.32	*plausible reporter*	under-reporter
USZO09	879.4	1234.1	2.13	0.71	under-reporter	under-reporter
USZO10	2354.6	1437.2	1.80	1.64	*plausible reporter*	*plausible reporter*
USZP01	1301.7	1320.7	1.59	0.99	under-reporter	under-reporter
USZP03	1090.5	1531.9	1.52	0.71	under-reporter	under-reporter
USZP05	620.6	1566.1	1.59	0.40	under-reporter	under-reporter
USZP06	1574.4	1462.4	1.65	1.08	under-reporter	under-reporter
USZP07	2047.1	1515.3	1.81	1.35	*plausible reporter*	under-reporter
USZP09	1704.6	1506.3	1.78	1.13	under-reporter	under-reporter
USZP10	843.5	1656.0	1.28	0.51	under-reporter	under-reporter
USZP11	1250.2	1304.4	1.88	0.96	under-reporter	under-reporter
USZP12	934.5	1428.5	1.50	0.65	under-reporter	under-reporter
USZP13	24HR not available					
USZP14	2201.4	1589.2	1.53	1.39	*plausible reporter*	under-reporter
USZP15	PAL not available					
USZP21	941.9	1453.0	1.12	0.65	under-reporter	under-reporter

* Classification with an S factor specific for the study population.
§ Classification with an S factor according to Black .[1]
BMR= adjusted basal metabolic rate. *PAL*=physical activity level. *REE*=resting energy expenditure by seca analytics 125. *TDEE*=total daily energy expenditure. *TDEI*=total daily energy intake.

Supplementary Table 23: Classification of study participants as plausible, over- or under-reporters of total daily energy intake estimated with SNAQ according to the Goldberg cut-off method,[1] using Resting Energy Expenditure (REE) calculated with seca analytics 125 using a proprietary regression model based on impedance-derived body composition, and Physical Activity Level (PAL) calculated as a ratio of DLW-based estimates of total daily energy expenditures (TDEE) and adjusted Basal Metabolic Rate (BMR) estimated by the Mifflin-St Jeor equation.

Study identifier	Energy Intake (kcal/day)	REE (kcal/day)	PAL (TDEE:BMR)	TDEI:REE	Classification (I)*	Classification (II)§
USZO02	PAL not available					
USZO04	1612.6	1723	2.1	0.94	under-reporter	under-reporter
USZO05	1158.0	1585	2.2	0.73	under-reporter	under-reporter
USZO06	4207.3	1540	1.7	2.73	*plausible reporter*	over-reporter
USZO08	1725.4	1681	2.3	1.03	under-reporter	under-reporter
USZO09	661.6	1558	2.7	0.42	under-reporter	under-reporter
USZO10	2730.7	1682	2.1	1.62	*plausible reporter*	under-reporter
USZP01	5562.3	1668	2.0	3.33	over-reporter	over-reporter
USZP03	2038.9	1776	1.8	1.15	under-reporter	under-reporter
USZP05	604.4	2191	2.2	0.28	under-reporter	under-reporter
USZP06	1879.3	1646	1.9	1.14	under-reporter	under-reporter
USZP07	2922.9	1945	2.3	1.50	under-reporter	under-reporter
USZP09	4862.9	1978	2.3	2.46	*plausible reporter*	over-reporter
USZP10	1208.7	2129	1.6	0.57	under-reporter	under-reporter
USZP11	1617.6	1695	2.4	0.95	under-reporter	under-reporter
USZP12	1199.7	1876	2.0	0.64	under-reporter	under-reporter
USZP13	3733.1	1702	2.9	2.19	*plausible reporter*	*plausible reporter*
USZP14	1512.0	1922	1.8	0.79	under-reporter	under-reporter
USZP15	PAL not available					
USZP21	2115.3	2070	1.6	1.02	under-reporter	under-reporter

* Classification with an S factor specific for the study population.
§ Classification with an S factor according to Black .[1]
BMR= adjusted basal metabolic rate. *PAL*=physical activity level. *REE*=resting energy expenditure by seca analytics 125. *TDEE*=total daily energy expenditure. *TDEI*=total daily energy intake.

Supplementary Table 24: Classification of study participants as plausible, over- or under-reporters of total daily energy intake estimated with the 24-hour dietary recall (24HR) according to the Goldberg cut-off method,[1] using Resting Energy Expenditure (REE) calculated with seca analytics 125 using a proprietary regression model based on impedance-derived body composition, and Physical Activity Level (PAL) calculated as a ratio of DLW-based estimates of total daily energy expenditures (TDEE) and adjusted Basal Metabolic Rate (BMR) estimated by the Mifflin-St Jeor equation.

Study identifier	Energy Intake (kcal/day)	REE (kcal/day)	PAL (TDEE:BMR)	TDEI:REE	Classification (I)*	Classification (II)§
USZO02	PAL not available					
USZO04	2272.3	1723	2.14	1.32	under-reporter	under-reporter
USZO05	835.9	1585	2.19	0.53	under-reporter	under-reporter
USZO06	1563.3	1540	1.68	1.02	under-reporter	under-reporter
USZO08	1953.8	1681	2.25	1.16	under-reporter	under-reporter
USZO09	879.4	1558	2.69	0.56	under-reporter	under-reporter
USZO10	2354.6	1682	2.11	1.40	under-reporter	under-reporter
USZP01	1301.7	1668	2.00	0.78	under-reporter	under-reporter
USZP03	1090.5	1776	1.76	0.61	under-reporter	under-reporter
USZP05	620.6	2191	2.22	0.28	under-reporter	under-reporter
USZP06	1574.4	1646	1.86	0.96	under-reporter	under-reporter
USZP07	2047.1	1945	2.32	1.05	under-reporter	under-reporter
USZP09	1704.6	1978	2.33	0.86	under-reporter	under-reporter
USZP10	843.5	2129	1.64	0.40	under-reporter	under-reporter
USZP11	1250.2	1695	2.45	0.74	under-reporter	under-reporter
USZP12	934.5	1876	1.97	0.50	under-reporter	under-reporter
USZP13	24HR not available					
USZP14	2201.4	1922	1.84	1.15	under-reporter	under-reporter
USZP15	PAL not available					
USZP21	941.9	2070	1.59	0.46	under-reporter	under-reporter

* Classification with an S factor specific for the study population.
§ Classification with an S factor according to Black .[1]
BMR= adjusted basal metabolic rate. *PAL*=physical activity level. *REE*=resting energy expenditure by seca analytics 125. *TDEE*=total daily energy expenditure. *TDEI*=total daily energy intake.

Supplementary Table 25: Classification of study participants as plausible, over- or under-reporters of total daily energy intake estimated with SNAQ according to the Goldberg cut-off method,[1] using Resting Energy Expenditure (REE) calculated with seca analytics 125 using a proprietary regression model based on impedance-derived body composition, and Physical Activity Level (PAL) selected based on lifestyle using the software seca analytics 125.

Study identifier	Energy Intake (kcal/day)	REE (kcal/day)	PAL (seca)	TDEI:REE	Classification (I)*	Classification (II)§
USZO02	PAL not available					
USZO04	1612.6	1723	1.6	0.94	under-reporter	under-reporter
USZO05	1158.0	1585	1.6	0.73	under-reporter	under-reporter
USZO06	4207.3	1540	1.6	2.73	over-reporter	over-reporter
USZO08	1725.4	1681	1.8	1.03	under-reporter	under-reporter
USZO09	661.6	1558	1.8	0.42	under-reporter	under-reporter
USZO10	2730.7	1682	1.0	1.62	*plausible reporter*	*plausible reporter*
USZP01	5562.3	1668	1.6	3.33	over-reporter	over-reporter
USZP03	2038.9	1776	1.6	1.15	under-reporter	under-reporter
USZP05	604.4	2191	1.8	0.28	under-reporter	under-reporter
USZP06	1879.3	1646	1.8	1.14	under-reporter	under-reporter
USZP07	2922.9	1945	1.4	1.50	*plausible reporter*	*plausible reporter*
USZP09	4862.9	1978	1.6	2.46	over-reporter	over-reporter
USZP10	1208.7	2129	1.8	0.57	under-reporter	under-reporter
USZP11	1617.6	1695	1.4	0.95	under-reporter	under-reporter
USZP12	1199.7	1876	1.8	0.64	under-reporter	under-reporter
USZP13	3733.1	1702	1.6	2.19	over-reporter	over-reporter
USZP14	1512.0	1922	1.4	0.79	under-reporter	under-reporter
USZP15	PAL not available					
USZP21	2115.3	2070	1.6	1.02	under-reporter	under-reporter

* Classification with an S factor specific for the study population.
§ Classification with an S factor according to Black .[1]
PAL=physical activity level. *REE*=resting energy expenditure by seca analytics 125. *TDEE*=total daily energy expenditure. *TDEI*=total daily energy intake.

Supplementary Table 26: Classification of study participants as plausible, over- or under-reporters of total daily energy intake estimated with the 24-hour dietary recall (24HR) according to the Goldberg cut-off method,[1] using Resting Energy Expenditure (REE) calculated with seca analytics 125 using a proprietary regression model based on impedance-derived body composition, and Physical Activity Level (PAL) selected based on lifestyle using the software seca analytics 125.

**Study identifier**	**Energy Intake (kcal/day)**	**REE (kcal/day)**	**PAL (seca)**	**TDEI:REE**	**Classification (I)***	**Classification (II)§**
USZO02	PAL not available					
USZO04	2272.3	1723	1.6	1.32	*plausible reporter*	under-reporter
USZO05	835.9	1585	1.6	0.53	under-reporter	under-reporter
USZO06	1563.3	1540	1.6	1.02	under-reporter	under-reporter
USZO08	1953.8	1681	1.8	1.16	under-reporter	under-reporter
USZO09	879.4	1558	1.8	0.56	under-reporter	under-reporter
USZO10	2354.6	1682	1.0	1.40	*plausible reporter*	under-reporter
USZP01	1301.7	1668	1.6	0.78	under-reporter	under-reporter
USZP03	1090.5	1776	1.6	0.61	under-reporter	under-reporter
USZP05	620.6	2191	1.8	0.28	under-reporter	under-reporter
USZP06	1574.4	1646	1.8	0.96	under-reporter	under-reporter
USZP07	2047.1	1945	1.4	1.05	under-reporter	under-reporter
USZP09	1704.6	1978	1.6	0.86	under-reporter	under-reporter
USZP10	843.5	2129	1.8	0.40	under-reporter	under-reporter
USZP11	1250.2	1695	1.4	0.74	under-reporter	under-reporter
USZP12	934.5	1876	1.8	0.50	under-reporter	under-reporter
USZP13	24HR not available					
USZP14	2201.4	1922	1.4	1.15	under-reporter	under-reporter
USZP15	PAL not available					
USZP21	941.9	2070	1.6	0.46	under-reporter	under-reporter

* Classification with an S factor specific for the study population.
§ Classification with an S factor according to Black .[1]
PAL=physical activity level. *REE*=resting energy expenditure by seca analytics 125. *TDEE*=total energy expenditure. *TDEI*=total daily energy intake.

Supplementary Table 27: Classification of study participants as plausible, over- or under-reporters of total daily energy intake estimated with SNAQ according to the Goldberg cut-off method,[1] using Resting Energy Expenditure (REE) calculated with seca analytics 125 using a proprietary regression model based on impedance-derived body composition, and Physical Activity Level (PAL) calculated as a ratio of DLW-based estimates of total daily energy expenditures (TDEE) and REE.

Study identifier	Energy Intake (kcal/day)	REE (kcal/day)	PAL (TDEE:REE)	TDEI:REE	Classification (I)*	Classification (II)§
USZO02	PAL not available					
USZO04	1612.6	1723	1.59	0.94	under-reporter	under-reporter
USZO05	1158.0	1585	1.88	0.73	under-reporter	under-reporter
USZO06	4207.3	1540	1.46	2.73	over-reporter	over-reporter
USZO08	1725.4	1681	1.98	1.03	under-reporter	under-reporter
USZO09	661.6	1558	2.13	0.42	under-reporter	under-reporter
USZO10	2730.7	1682	1.80	1.62	*plausible reporter*	*plausible reporter*
USZP01	5562.3	1668	1.59	3.33	over-reporter	over-reporter
USZP03	2038.9	1776	1.52	1.15	under-reporter	under-reporter
USZP05	604.4	2191	1.59	0.28	under-reporter	under-reporter
USZP06	1879.3	1646	1.65	1.14	under-reporter	under-reporter
USZP07	2922.9	1945	1.81	1.50	*plausible reporter*	under-reporter
USZP09	4862.9	1978	1.78	2.46	over-reporter	over-reporter
USZP10	1208.7	2129	1.28	0.57	under-reporter	under-reporter
USZP11	1617.6	1695	1.88	0.95	under-reporter	under-reporter
USZP12	1199.7	1876	1.50	0.64	under-reporter	under-reporter
USZP13	3733.1	1702	2.44	2.19	*plausible reporter*	over-reporter
USZP14	1512.0	1922	1.53	0.79	under-reporter	under-reporter
USZP15	PAL not available					
USZP21	2115.3	2070	1.12	1.02	under-reporter	under-reporter

* Classification with an S factor specific for the study population.
§ Classification with an S factor according to Black .[1]
PAL=physical activity level. *REE*=resting energy expenditure by seca analytics 125. *TDEE*=total daily energy expenditure. *TDEI*=total daily energy intake.

Supplementary Table 28: Classification of study participants as plausible, over- or under-reporters of total daily energy intake estimated with the 24-hour dietary recall (24HR) according to the Goldberg cut-off method,[1] using Resting Energy Expenditure (REE) calculated with seca analytics 125 using a proprietary regression model based on impedance-derived body composition, and Physical Activity Level (PAL) calculated as a ratio of DLW-based estimates of total daily energy expenditures (TDEE) and REE.

Study identifier	Energy Intake (kcal/day)	REE (kcal/day)	PAL (TDEE:REE)	TDEI:REE	Classification (I)*	Classification (II)§
USZO02	PAL not available					
USZO04	2272.3	1723	1.59	1.32	*plausible reporter*	under-reporter
USZO05	835.9	1585	1.88	0.53	under-reporter	under-reporter
USZO06	1563.3	1540	1.46	1.02	under-reporter	under-reporter
USZO08	1953.8	1681	1.98	1.16	under-reporter	under-reporter
USZO09	879.4	1558	2.13	0.56	under-reporter	under-reporter
USZO10	2354.6	1682	1.80	1.40	*plausible reporter*	under-reporter
USZP01	1301.7	1668	1.59	0.78	under-reporter	under-reporter
USZP03	1090.5	1776	1.52	0.61	under-reporter	under-reporter
USZP05	620.6	2191	1.59	0.28	under-reporter	under-reporter
USZP06	1574.4	1646	1.65	0.96	under-reporter	under-reporter
USZP07	2047.1	1945	1.81	1.05	under-reporter	under-reporter
USZP09	1704.6	1978	1.78	0.86	under-reporter	under-reporter
USZP10	843.5	2129	1.28	0.40	under-reporter	under-reporter
USZP11	1250.2	1695	1.88	0.74	under-reporter	under-reporter
USZP12	934.5	1876	1.50	0.50	under-reporter	under-reporter
USZP13	24HR not available					
USZP14	2201.4	1922	1.53	1.15	under-reporter	under-reporter
USZP15	PAL not available					
USZP21	941.9	2070	1.12	0.46	under-reporter	under-reporter

* Classification with an S factor specific for the study population.
§ Classification with an S factor according to Black .[1]
PAL=physical activity level. *REE*=resting energy expenditure by seca analytics 125. *TDEE*=total daily energy expenditure. *TDEI*=total daily energy intake.

Supplementary Table 29: Summary of the number of plausible, under-, and over-reporters across all combinations of BMR, REE, and PAL estimation methods for the SNAQ and 24-hour dietary recall (24HR), calculated using both a study-specific S factor and the reference values proposed by Black.[1]

Estimation method	PAL derivation	Dietary assessment tool	N	S factor (specific for the study population)			S factor (according to Black [1])		
				n below	n plausible	n above	n below	n plausible	n above
**aBMR (Mifflin-St Jeor)**	PAL (TDEE:BMR)	SNAQ	20	14	3	3	15	1	4
		24HR	19	17	2	0	19	0	0
	PAL (seca)	SNAQ	18	7	5	4	12	0	6
		24HR	17	12	3	0	15	1	1
	**PAL (TDEE:REE)**	**SNAQ**	**18**	**8**	**6**	**4**	**12**	**0**	**6**
		24HR	17	12	5	0	15	2	0
REE (seca)	PAL (TDEE:BMR)	SNAQ	18	13	4	1	14	1	3
		24HR	17	17	0	0	17	0	0
	PAL (seca)	SNAQ	18	12	2	4	12	2	4
		24HR	17	15	2	0	17	0	0
	PAL (TDEE:REE)	SNAQ	18	12	3	3	13	1	4
		24HR	17	15	2	0	17	0	0

BMR=adjusted basal metabolic rate. *PAL*=physical activity level. *REE*=resting energy expenditure by seca analytics 125. *TDEE*=total daily energy expenditure. *TDEI*=total daily energy intake.

Supplementary Table 30: Results of the Bland-Altman plot for agreement between bioelectrical impedance (BIA) and isotope dilution of DLW for measurements of body composition

Agreement between:	Bias	lCI bias	uCI bias	SD bias	SE bias	lLoA	uLoA	SE LoA	lCI lLoA	uCI lLoA	lCI uLoA	uCI uLoA
TBW	−1.3	−1.7	4.4	6.2	1.5	−10.9	13.5	2.5	−16.2	−5.5	8.2	18.9
FFM	0.5	−3.7	4.6	8.3	1.9	−15.8	16.7	3.9	−22.9	−8.6	9.6	23.8
FM	−0.9	−4.7	3.0	7.8	1.8	−16.1	14.3	3.2	−22.7	−9.4	7.7	21.0

TBW, FFM, and FM were calculated in kg. DLW was selected as reference method for the analysis.
DLW=doubly labelled water. TBW=Total Body Water. FFM=Fat-Free Mass. FM=Fat Mass.
Bias=bias of agreement. lCI=lower 95 % confidence interval. lLoA=lower limit of agreement. SD=standard deviation. SE=standard error.
uCI=upper 95 % confidence interval. uLoA=upper limit of agreement.

References

1. Black, A.E., *Critical evaluation of energy intake using the Goldberg cut-off for energy intake: basal metabolic rate. A practical guide to its calculation, use and limitations.* International journal of obesity, 2000. **24**(9): p. 1119-1130.
